# The *achaete*‐*scute* complex in Diptera: patterns of noncoding sequence evolution

**DOI:** 10.1111/jeb.12687

**Published:** 2015-09-07

**Authors:** B. Negre, P. Simpson

**Affiliations:** ^1^ Department of ZoologyUniversity of CambridgeCambridgeUK; ^2^ Present address: Departament de Genètica i de MicrobiologiaUniversitat Autònoma de Barcelona08193Bellaterra(Barcelona)Spain

**Keywords:** achaete‐scute complex, *Calliphora*, Diptera, *Drosophila*, gene duplication, regulatory elements, sequence evolution

## Abstract

The *achaete‐scute* complex (AS‐C) has been a useful paradigm for the study of pattern formation and its evolution. *achaete‐scute* genes have duplicated and evolved distinct expression patterns during the evolution of cyclorraphous Diptera. Are the expression patterns in different species driven by conserved regulatory elements? If so, when did such regulatory elements arise? Here, we have sequenced most of the AS‐C of the fly *Calliphora vicina* (including the genes *achaete*,* scute* and *lethal of scute*) to compare noncoding sequences with known *cis‐*regulatory sequences in *Drosophila*. The organization of the complex is conserved with respect to *Drosophila* species. There are numerous small stretches of conserved noncoding sequence that, in spite of high sequence turnover, display binding sites for known transcription factors. Synteny of the blocks of conserved noncoding sequences is maintained suggesting not only conservation of the position of regulatory elements but also an origin prior to the divergence between these two species. We propose that some of these enhancers originated by duplication with their target genes.

## Introduction

Most genes originate by gene duplication. When a gene duplicates, it will eventually have one of three possible outcomes: loss, subfunctionalization (the original function is divided between the two new copies) or neofunctionalization (the daughter gene acquires a new function) (Force *et al*., 1999). The acquisition of new functions can be due to the evolution of the protein itself or to the acquisition of new domains of expression (in both space and time). Many of these gene duplications occur in tandem, giving rise to groups of related genes. The combination of gene duplication and subfunctionalization is thus at the origin of gene complexes: groups of paralogous genes with related functions. Some examples are the Hox complex, the *achaete‐scute* complex (AS‐C), the Iroquois complex, or the gene pairs *engrailed*/*invected* and *eyegone*/*twin of eyegone*, most of which are widely conserved. It is this widespread conservation that has been interpreted as a necessity for proper function. But is the conserved structure always necessary? In some cases, it has been suggested that it is the existence of shared regulatory elements that prevent the separation of the genes. However, we know very little about how regulatory elements originate and evolve. Here, we examine the AS‐C of two different fly species to identify regulatory elements and obtain insights about their origin and evolution.

**Figure 1:**
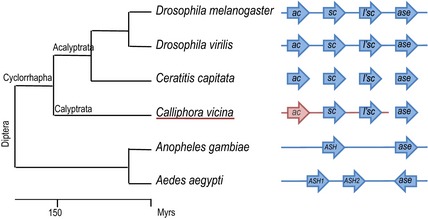
Dipteran phylogeny showing the available information about the AS‐C. Arrows indicate Dipteran coding genes. Genes are connected with a line when genome organization is known. Previously published data are shown in blue, data obtained in this work in red.

The AS‐C is a good example of gene duplication and subfunctionalization (Negre & Simpson, 2009). Originally described in *Drosophila*,* achaete‐scute homologue* (*ASH*) genes are present in all metazoans and have undergone independent duplication in different lineages (Fig. 1; Negre & Simpson, 2009). The AS‐C has been studied extensively; it is a model for the study of development and pattern formation. It is involved in neural development and the specification of sensory organs (as for example, fly bristles).

The *Drosophila melanogaster* AS‐C has four genes that encode transcriptional regulators of the basic helix‐loop‐helix family (Campuzano *et al*., 1985; Villares & Cabrera, 1987; Alonso & Cabrera, 1988; Gonzalez *et al*., 1989): three are proneural genes *achaete* (ac), *scute* (*sc*) and *lethal of scute* (*l'sc*) and the fourth a neural precursor gene *asense* (*ase*) (Fig. 1). All four genes are devoid of introns, show the same orientation and are clustered in a 100‐kb region containing numerous shared and interspersed *cis‐*regulatory elements (Gomez‐Skarmeta *et al*., 1995). The genes *yellow* (*y*) and *CytP450* delimit the ends of this gene complex. Genetic rearrangements within the complex generally lead to mutant phenotypes due to disruption of *cis‐*regulatory organization (Ruiz‐Gomez & Modolell, 1987; Ghysen & Dambly‐Chaudiere, 1988). Thus, it is generally thought that the organization of the complex and the presence of shared *cis‐*regulatory regions prevent separation of the genes.

The ancestral AS‐C in insects was composed of two genes: an *achaete‐scute homologue* (*ASH*) gene and an *ase* gene (Negre & Simpson, 2009; Ayyar *et al*., 2010). The proneural gene *ASH* has undergone independent duplications in different lineages. Coleoptera and Hymenoptera show the ancestral configuration with one *ASH* and one *ase* gene. The genes are clustered and surrounded by *CytP450* and *yellow* genes as in *Drosophila* (except that *Tribolium* lacks *yellow*). The lepidopteran, *Bombyx*, has one *ase* and three *ASH* genes, which represent independent duplications from those of Diptera (Negre & Simpson, 2009). The genes *yellow* and *CytP450* also delimit the edges of the gene complex. Mosquitoes bear one *ase* gene and one or two *ASH* genes, a duplication that occurred after the split of the *Aedes/Culex* and *Anopheles* lineages (see Fig. 1). In all three mosquitoes, the genes *yellow* and *CytP450* are associated with the AS‐C genes. Finally, the 12 *Drosophila* genomes show the same configuration as *D. melanogaster*, with three *ASH* genes (*ac*,* sc* and *l'sc*) and one *ase* gene in the same order and orientation, in all but one species the gene complex is also delimited by the genes *yellow* and *CytP450*. Although we have some information on the genes present and expression patterns of other Cyclorraphous Diptera, there is no information on whether the genes are clustered or of their regulatory elements. All insects examined so far have one *ase* gene and one or several *ASH* genes.

The increase in number of *ASH* genes has occurred independently in different lineages and correlates with morphological diversification in both flies and butterflies. In butterflies, *ASH* duplications could be related to the origin or differentiation of wing scales (Negre & Simpson, 2009). In flies, *ASH* duplications correlate with the emergence and patterning of macrochaetes. The Nematocera do not bear macrochaetes and the notum is generally uniformly covered with bristles, implying that there would be little need of spatial resolution of proneural gene expression (McAlpine, 1981; Simpson *et al*., 1999). Indeed, *Anopheles gambiae* bears only two genes in the AS‐C, whereas two tandem duplications in the lineage leading to the Cyclorrapha have led to a total of four genes in the AS‐C of *Drosophila* and *Musca domestica* (Skaer *et al*., 2002; Wulbeck & Simpson, 2002; Wrischnik *et al*., 2003).

The four genes of *Drosophila*,* ac*,* sc*,* l'sc* and *ase*, have undergone subfunctionalization (Force *et al*., 1999). They are regulated by both shared and independent regulatory elements (Jarman *et al*., 1993; Gomez‐Skarmeta *et al*., 1995; Culi & Modolell, 1998; Wrischnik *et al*., 2003). Presumably these elements have arisen during the evolution of cyclorraphous flies (Simpson & Marcellini, 2006). Multiple studies have tried to dissect the regulatory elements of the *Drosophila* AS‐C. Regions corresponding to expression in the embryonic nervous system and to an early enhancer involved in sex determination have been roughly characterized (Skeath *et al*., 1992; Wrischnik *et al*., 2003). A number of regions driving co‐expression of *ac* and *sc* in the wing disc were functionally defined (Gomez‐Skarmeta *et al*., 1995, 1996; Garcia‐Garcia *et al*., 1999). They were initially identified by virtue of the phenotypic effects of breakpoints within the AS‐C and regions of hybridization between AS‐C DNA of *D. melanogaster* and *Drosophila virilis* (Ruiz‐Gomez & Modolell, 1987; Gomez‐Skarmeta *et al*., 1995). Rather few of these regulatory modules have been defined as minimal enhancers with known (tested) transcription factor binding sites. These are the DorsoCentral Enhancer (DCE), the Sensory Organ Precursor Enhancer (SOPE) and the L3/TSM enhancer. One possibility is that duplication of regulatory sequences accompanied duplication of coding sequences and that the regulatory elements subsequently diverged. The upstream transcriptional regulators of *ac‐sc* appear to be conserved, so much of the evolution is likely to have occurred in *cis*, at the level of AS‐C regulatory sequences (Richardson & Simpson, 2006). Indeed, there is one regulatory element with divergent expression patterns between drosophilid species (Marcellini & Simpson, 2006).

Highly conserved noncoding sequences have been identified in diverse vertebrate species and have led to the identification of long‐range enhancers in developmental genes (Bejerano *et al*., 2004; Siepel *et al*., 2005; Woolfe *et al*., 2005). Some of these can be traced back to the origins of vertebrates, 500 Myr ago (McEwen *et al*., 2009). This approach has been less successful for invertebrate genomes, where few regulatory elements have been functionally identified other than between quite closely related species. However, numerous small stretches of conserved noncoding DNA with conserved synteny are found within drosophilids and between drosophilids and *A. gambiae* and even between more distantly related insects (Zdobnov *et al*., 2002; Glazov *et al*., 2005; Papatsenko *et al*., 2006; Engstrom *et al*., 2007; Zdobnov & Bork, 2007). One ancient regulatory module, found in *ase*, has been traced back to the last common ancestor of the Arthropoda, 550 Myr ago (Ayyar *et al*., 2010). It was identified by virtue of its conserved location in the UTR of *ase* and would not have been detected on the basis of sequence alignment. The study of specific enhancers between dipteran species has demonstrated rapid turnover of regulatory sequences in spite of conservation of function (Bonneton *et al*., 1997; Ludwig *et al*., 1998, 2000, 2005; McGregor *et al*., 2001; Ludwig, 2002; Wittkopp, 2006; Wratten *et al*., 2006; Hare *et al*., 2008).

It has been suggested that vertebrates differ from invertebrates by virtue of their large genomes in which small stretches of conserved noncoding sequences are interspersed with large stretches of nonconserved DNA, a feature that facilitates detection of conserved sequences (Peterson *et al*., 2009). Indeed, one study examined early patterning genes of four species of Tephritidae, a family diverged from Drosophilidae by about 100 Myr and containing species with significantly larger genomes. It revealed small blocks of conserved sequence among large stretches of poor conservation (Peterson *et al*., 2009). Furthermore, a study of six species of Sepsidae found that two‐thirds of conserved blocks from the *even‐skipped* gene were functional (Hare *et al*., 2008). In addition, numerous short stretches of sequence were similar to the corresponding regions of *D. melanogaster*, especially those enriched in pairs of overlapping or adjacent binding sites (Hare *et al*., 2008). This suggests that detection of conserved sequence blocks in *Calliphora vicina*, a species with a much larger genome than *Drosophila*, might be helpful for the identification of regulatory elements.

*Calliphora sc*,* l'sc* and *ase* genes have been cloned, and the timing and tissue specificity of their expression patterns are equivalent to those of *Drosophila* (Pistillo *et al*., 2002). However, we have no information about their genomic organization or their regulatory sequences. Are expression patterns in *Drosophila* and *Calliphora* driven by the same regulatory elements? When did these regulatory elements arise? Do they correlate with gene duplications?

The aim of this study was to sequence the region of the *ac‐sc* genes of *C. vicina* in order to examine the degree of conservation of AS‐C architecture and to identify regulatory sequences. *C. vicina* probably diverged from *D. melanogaster* about 150 Myr ago and has a much bigger genome than the latter. We have isolated and sequenced most of the AS‐C of *C. vicina*. We find conservation of the overall structure of the complex including coding and noncoding DNA. We suggest that some regulatory modules predate the divergence between the *Drosophila* and *Calliphora* lineages and originated by gene duplication with their target gene.

## Materials and methods

### Flies

A *C. vicina* nonhomogenized strain was used for the construction of a genomic BAC library (details below) and for degenerate PCR. The size of the *C. vicina* genome is 750 Mb as estimated by flow cytometry from this strain (S. Jonhston, personal communication).

### PCR amplification

A fragment of the *yellow* (*y*) gene from *C. vicina* was obtained by degenerate PCR from genomic DNA with the following primers: F: TGGGARCARAAYAARWSITGG; R: TGCCARCANCCNACNGCRTT. Temperature cycling conditions were 35 cycles of 40 s at 94 °C, 40 s at 45.5 °C and 90 s at 72 °C. The PCR product was cloned into pGEM‐Teasy and sequenced.

### *Calliphora vicina* BAC library and library screening

We used an arrayed BAC library of *C. vicina* with 6.8× coverage (constructed by Amplicon Express, Pullman, WA, USA). High molecular weight genomic DNA was obtained from frozen starved third‐instar larvae. DNA was partially digested with *Hin*dIII, size‐fractioned and cloned in the pCC1BAC vector (Epicentre). The arrayed library contains 46 080 clones with an average insert size of 115 kb. The library was spotted in high‐density colony filters for screening purposes.

**Figure 2:**
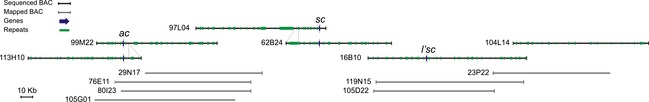
Map of the AS‐C region in *Calliphora vicina*. Sequenced BACs are shown in black and other BACs in grey. Blue arrows represent AS‐C genes and green boxes transposable elements and repeats.

The BAC library was screened with digoxigenin labelled probes following standard protocols. Fragments of *sc*,* l'sc*,* ase*,* y*,* ac*, and noncoding sequences from BAC ends were used as probes. Probes were hybridized in pools of 2–5 probes. Positive clones were confirmed by PCR. Additional PCRs with probe fragments and other sequences (e.g. BAC ends) were used to construct a physical map of the region. All 13 positive clones form a single contig (Fig. 2). Six clones covering the whole region were selected for sequencing (Fig. 2, Table 1). Selected BAC clones were subcloned and sequenced by the Sanger method at Amplicon Express and assembled with SeqMan (Lasergene package, DNAstar, Madison, WI, USA).

**Table 1:** Summary of sequenced BAC clones. Gene and repeat content

BAC	Total size (bp)	Genes			Repeats		
		Number	bp	%	Number	bp	%
113H10	96 426	1 (*ac*)	885	0.92	61	23 570	24.44
99M22	102 758	1 (*ac*)	885	0.86	78	28 879	28.10
97L04	111 044	1 (*sc*)	963	0.87	38	38 432	34.61
62B24	90 178	1 (*sc*)	963	1.07	44	28 013	31.06
16B10	135 393	1 (*l'sc*)	786	0.58	66	27 144	20.05
104L14	115 595	0	0	0	68	19 608	16.96
Total	651 394	5	4482	0.69	355	165 646	25.43
Without overlap	530 000	3	2634	0.50			

### Sequence annotation

BAC sequences were annotated manually in Artemis (Berriman & Rutherford, 2003). Sequences were compared to the protein database (by blastx) and to the nonredundant (nr) nucleotide database (by blastn). Only three fly genes were detected: *sc*,* l'sc* and *ac*. All other hits correspond to transposases, retrotranscriptases and other repeat sequences (described in Negre & Simpson, 2013).

The accession numbers to the sequences described in the paper are LN877230‐LN877236.

### Detection of conserved sequences

BAC clone sequences from *C. vicina* were compared with the AS‐C from *D. melanogaster* (ChrX 210 000–330 000) and *D. virilis* with blast2sequences and mVISTA (Frazer *et al*., 2004). Blast2sequence hits with *e*‐value lower than 0.1 were selected (Table 2). We discarded several hits within coding regions and four that corresponded to repeats or TEs. Alignments corresponding to selected hits are shown in Fig. S2.

**Table 2:** Conserved noncoding sequences of the achaete‐scute complex detected by blast2seq between *Calliphora* and *Drosophila*

*Drosophila melanogaster*		*Calliphora vicina*			Blast2sequences hits					
Start	End	Clone	Start	End	% Identity	Length	Mismatches	Gap opens	*e*‐Value	Bit score
13 423	13 479	113H10	18 171	18 211	71.93	57	0	2	0.066	37.4
14 226	14 304	113H10	20 773	20 851	72.62	84	13	3	4.00E‐04	44.6
19 665	19 697	99M22	37 506	37 474	90.91	33	3	0	1.00E‐04	46.4
23 323^7^	23 352	99M22	57 812	57 841	93.33	30	2	0	1.00E‐04	46.4
25 810^7^	25 845	99M22	73 435	73 400	100.00	36	0	0	1.00E‐10	66.2
33 788	33 828	97L04	44 884	44 925	90.48	42	3	1	3.00E‐07	55.4
34 886	34 928	97L04	50 672	50 630	90.70	43	4	0	7.00E‐09	60.8
37 687	37 713	97L04	24 868	24 894	96.30	27	1	0	5.00E‐04	44.6
38 125	38 149	97L04	70 125	70 101	96.00	25	1	0	0.006	41.0
40 105^1^	40 129	97L04	90 093	90 069	92.00	25	2	0	0.076	37.4
		*62B24*	*14* *103*	*14* *079*					*0.062*	
42 356^2^	42 403	97L04	99 269	99 316	89.58	48	5	0	5.00E‐10	64.4
		*62B24*	*21* *655*	*21* *702*					*4*.*00E‐10*	
42 858^5^	42 923	97L04	104 855	104 920	81.82	66	12	0	2.00E‐10	66.2
		*62B24*	*27* *740*	*27* *805*					*1*.*00E‐10*	
46 110	46 141	62B24	47 973	47 942	90.62	32	3	0	4.00E‐04	44.6
46 525	46 558	62B24	46 965	46 931	91.43	35	2	1	1.00E‐04	46.4
51 057^3^	51 103	62B24	72 470	72 424	87.23	47	6	0	2.00E‐08	59.0
		*16B10*	*2419*	*2373*					*3*.*00E‐08*	
51 964^3^	52 013	16B10	30 646	30 599	86.00	50	5	1	1.00E‐07	57.2
52 755^3^	52 844	16B10	24 209	24 116	82.98	94	12	3	6.00E‐17	87.8
53 731^3^	53 762	62B24	89 286	89 255	93.75	32	2	0	1.00E‐05	50.0
		*16B10*	*19* *235*	*19* *204*						
57 555^6^	57 671	16B10	50 662	50 779	70.25	121	29	4	6.00E‐04	44.6
66 739^8^	66 764	16B10	117 858	117 833	92.31	26	2	0	0.026	39.2
68 213^8^	68 243	104L14	28 982	29 012	90.32	31	3	0	0.002	42.8
68 286^8^	68 311	104L14	29 081	29 106	92.31	26	2	0	0.023	39.2
69 400^8^	69 423	104L14	32 865	32 888	95.83	24	1	0	0.023	39.2
69 638^8^	69 686	104L14	32 987	33 035	86.00	50	5	2	4.00E‐06	51.8
72 901^4^	72 925	104L14	26 919	26 895	100.00	25	0	0	2.00E‐04	46.4
86 922	86 977	104L14	54 520	54 575	87.50	56	7	0	1.00E‐11	69.8
90 692^9^	90 719	104L14	69 009	69 036	100.00	28	0	0	4.00E‐06	51.8
109 968^10^	109 997	97L04^a^	107 083	107 054	86.67	30	4	0	0.076	37.4

Fragments detected when comparing the *C. vicina* sequence to both *D. melanogaster* and *Drosophila virilis* (see text for details). The coordinates for *D. melanogaster* refer to the sequence ChrX 210 000–330 000 from the whole genome sequence. The *C. vicina* coordinates refer to each BAC clone (italics: fragments present in more than one BAC clone). Fragments overlapping known structures in *D. melanogaster*: wing enhancers ^1^sc‐SOPE, ^2^L3/TSM, ^3^pTG, ^4^tr1‐tr2; UTRs ^5^sc ^6^l'sc; genetically inferred blastoderm enhancers ^7^A, ^8^C, ^9^D, ^10^E.

a Worst hits (high *e*‐value in addition to short length or low % identity), and these are shown with a thin line in Fig. 2.

mVISTA pairwise alignments were performed with AVID (Bray *et al*., 2003), LAGAN (Brudno *et al*., 2003a) and Shuffle‐LAGAN (Brudno *et al*., 2003b) algorithms. CNS were detected with three parameter sets for each algorithm: (i) default parameters (minimum conserved width = 100 bp, conserved identity = 70%), (ii) minimum conserved width = 25 bp, conserved identity = 85% and (iii) minimum conserved width = 15 bp, conserved identity = 95%. Tables of the conserved blocks identified can be found in Table S1.

### Sequence alignments

ClustalW (Larkin *et al*., 2007) was used for sequence alignments between selected fragments (< 1 kb). Protein alignments were performed with T‐Coffee (Notredame *et al*., 2000; Di Tommaso *et al*., 2011) using default parameters.

## Results

### The AS‐C from *Calliphora vicina*

In the present study, we searched a *Calliphora* BAC library with fragments of *sc*,* l'sc*,* ase* and *y*. Coding sequence for three genes, *sc*,* l'sc* and *ase*, had been previously isolated from *C. vicina* by degenerate PCR, but it was not known whether they are grouped (Pistillo *et al*., 2002). We obtained a fragment of the *y* gene from *C. vicina* by degenerate PCR (see Methods). The library screening yielded two clones containing *sc* and three *l'sc*. No clones for the *y* or *ase* regions were obtained. We used sequences of BAC ends from *sc* and *l'sc* positive clones to further screen the library. These screenings yielded eight additional clones. The 13 positive BAC clones obtained were mapped into one single contig (Fig. 2). Six clones covering the whole contig were selected for sequencing (Fig. 2, Table 1). The six sequenced clones add up to a total of 651 394 bp (Table 1). The clones overlap 38 828 bp of identical sequence corresponding to the same allele found in different clones, as well as 83 000 bp corresponding to different alleles of the same region. Overall, the region sequenced covers 530 kb of the genome.

The region sequenced contains coding sequence for three fly genes *sc*,* l'sc* and also *ac*, which had not been cloned in this species. All three genes are intronless and show the same orientation. All other ORFs correspond to transposable elements; these elements, which account for 24% of the sequence, have been described in detail in Negre & Simpson (2013). *ac*,* sc* and *l'sc* are grouped in a gene complex. Although we have sequenced 82‐kb upstream of *ac* and 190‐kb downstream of *l'sc*, neither *ase* nor *pcl* nor the genes flanking the complex were reached.

The size of the gene complex was expected to be proportional to the increase in genome size, as has been observed for the AS‐C of drosophilids (our observations) and also for other loci in Diptera (Peterson *et al*., 2009). *D. melanogaster* has a 104 kb AS‐C and a 176 Mb genome, and the *C. vicina* genomes is 750 Mb: we estimated that the *C. vicina* AS‐C complex would be around 452 kb long. However, comparison of the noncoding sequences with those of the *D. melanogaster* AS‐C (see below) suggests that the 530 kb sequenced correspond to approximately 78% of the complex, and with these data, we now estimate the *C. vicina* AS‐C is 660 kb. Therefore, the complex is 50% larger than expected, and about 130 kb of the *C. vicina* AS‐C is probably still missing.

### Detection of conserved noncoding sequences

We aligned each BAC sequence with the *D. melanogaster* AS‐C to see whether there was conservation in noncoding sequences. We used two approaches to detect sequence conservation: global alignment with mVISTA and search of short conserved fragments with blast. In mVISTA, we used all three alignment algorithms available and three parameter sets to detect conserved blocks (see Methods and Table S1). We obtain very similar results in AVID and MLAGAN alignments. Only coding sequences are detected with default parameters (100 bp 70% id). Six conserved noncoding sequences (CNS) are detected when looking for short and highly conserved fragments (25 bp 85% or 15 bp 95%) in both alignments. The same number of fragments is identified in each alignment regardless of the parameters used, but only one coincides between alignments. Up to 12 CNS are detected in the SLAGAN alignment, as this algorithm allows for sequence rearrangements. It finds the same CNS as MLAGAN and six additional ones in putative rearranged regions.

To overcome the limitations of global alignment tools and because blast is able to detect short conserved sequences (e.g. containing two binding sites) independently of their orientation, we used blast2sequences‐blastn to compare noncoding sequences of *C. vicina* and *D. melanogaster*. We selected hits with an *e*‐value lower than 0.1 (Table 2, Table S1). Four correspond to repeats in *C. vicina* and were discarded. Forty‐three conserved blocks of sequence were detected, interspersed by regions of poor conservation. Most comprise a few tens of base pairs. To verify their significance, we extended the comparison to the *D. virilis* AS‐C. Twenty‐eight of the same sequence blocks were recovered (Table 2), suggesting that they have been retained through selection and are truly homologous.

**Figure 3:**
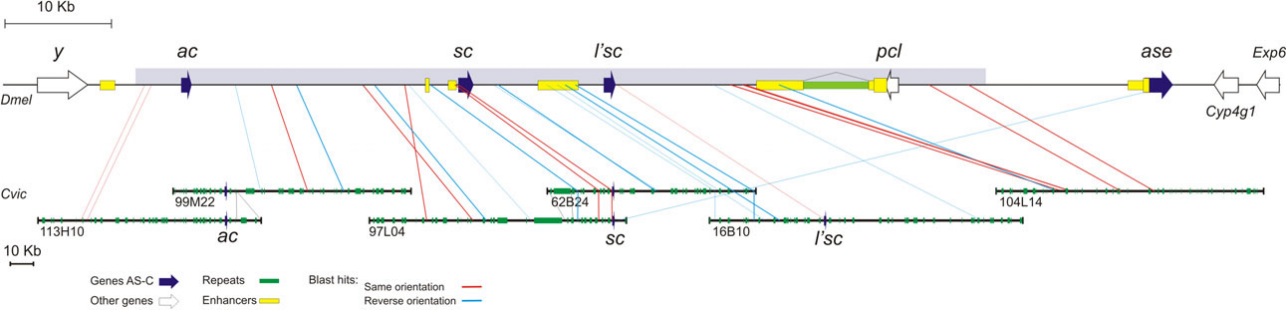
Comparison of AS‐C noncoding sequences between *Calliphora vicina* and *Drosophila melanogaster*. Note the different scale in each species. Arrows represent genes: AS‐C genes in blue, other genes in white. Green boxes indicate repeats and yellow boxes enhancers tested in *D. melanogaster*. The shadowed area in the *D. melanogaster* AS‐C indicates the approximate area included in the *C. vicina* sequence. Blue and red lines indicate conserved noncoding sequences detected between *C. vicina* and *D. melanogaster* and *Drosophila virilis* (details in Table 2).

Although the amount of sequence conservation is very low, the conserved fragments in *D. virilis* are also colinear between *D. melanogaster*/*D. virilis* and *C. vicina*. The relative position of the conserved sequence blocks between the three species is shown in Fig. 3. There is significant correlation between species, the order of the blocks being largely maintained. Some blocks change orientation (microinversions). Only one hit does clearly break colinearity (Table 2, Fig. 3). As the conserved sequences span 78% of the *D. melanogaster* AS‐C, we estimate that the region we have sequenced in *C. vicina* corresponds to approximately 78% of the AS‐C (see Fig. 3).

### Identification of putative enhancer sequences of the *Calliphora vicina* AS‐C

We compared the location of the blast2sequences hits with the sites of known enhancers in *D. melanogaster*. We found that 22 of the 28 blocks correlate with the positions of enhancers: seven with wing disc enhancers, two with UTRs of *sc* and *l'sc* and 13 with embryonic nervous system enhancers. We have further analysed the enhancers for which we have information of specific binding sites: the *L3/TSM* and the *SOP* enhancers.

**Figure 4:**
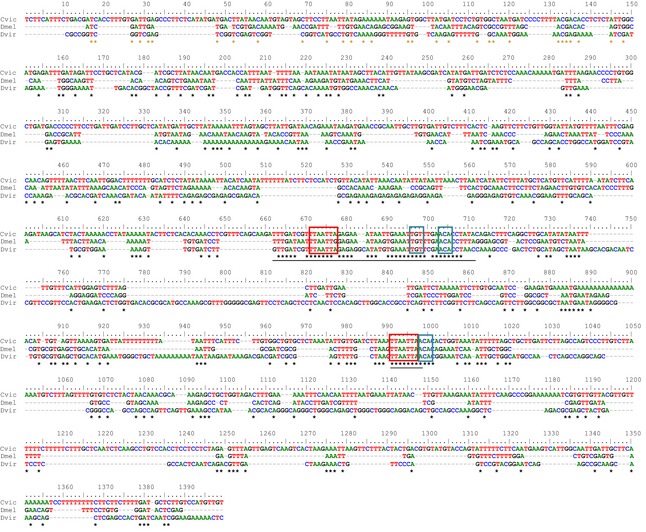
Sequence alignment of the L3/TSM enhancer. ClustalW2 alignment of the L3/TSM enhancer region between *Drosophila melanogaster* (Dmel), *Drosophila virilis* (Dvir) and *Calliphora vicina* (Cvic). Underlined are the blast2sequences hit (see Table 2 for details) and the TTAATTAA homeobox binding site identified by Gomez‐Skarmeta *et al*. (1996). Red boxes are En/Antp binding sites and blue boxes Ara/Caup binding sites, as defined by Noyes *et al*. (2008).

There is one blast2seq hit in the region of the *L3/TSM* enhancer (see Figs 3 and 4, Table 2). The *L3/TSM* enhancer of *D. melanogaster* drives expression of *ac* and *sc* in two regions on the wing where sensilla campaniforma arise: on the third longitudinal vein (L3) and the twin sensilla of the margin (TSM) (Gomez‐Skarmeta *et al*., 1996). These sensilla campaniforma are also present in *C. vicina* at homologous positions (Dickinson & Palka, 1987; Dickinson *et al*., 1997). Activation of the *ac* and *sc* promoters by this enhancer is mediated by the Iroquois homeobox proteins Araucan (Ara) and Caupolican (Caup). Gomez‐Skarmeta *et al*. (1996) identified a sequence, TTAATTAA (which corresponds to a homeobox binding site), as required for the activity of the L3/TSM enhancer and identified it as the Ara/Caup binding site. Recent analyses, however, have redefined the Ara/Caup binding site as ACA and the sequence TTAATTAA as a binding site of homeobox proteins of the En/Antp group (Noyes *et al*., 2008). The conserved sequence (blast hit) has two potential Iro binding sites and one En/Antp group binding site (as defined in Noyes *et al*., 2008). It is also in close proximity to the TTAATTAA sequence identified by Gomez‐Skarmeta *et al*. (1996), which corresponds to an En/Antp binding site overlapping an Iro binding site (Fig. 4). Thus, the enhancer is possibly dependent on the activation by an En/Antp homeodomain protein, in addition to Ara/Caup. The core sequence of this enhancer is conserved between *Drosophila* and *Calliphora*.

**Figure 5:**
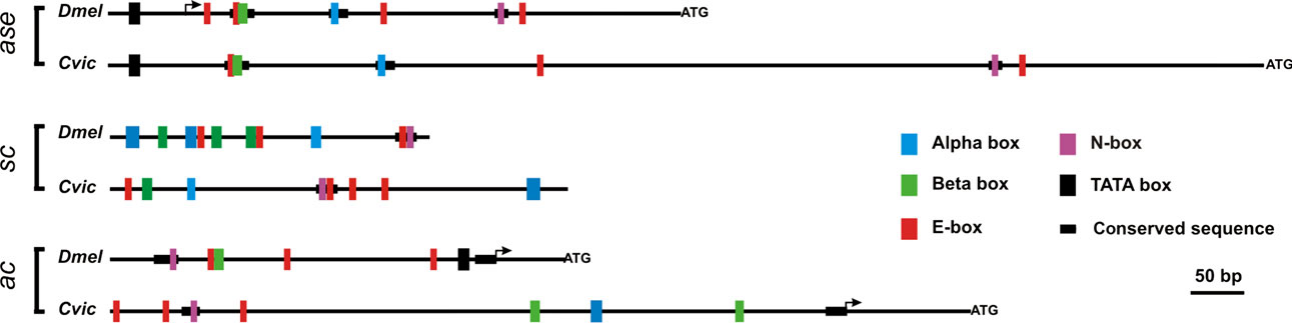
Comparison of the structure of the *SOP* enhancers of *asense*,* scute* and *achaete* between *Drosophila melanogaster* and *Calliphora vicina*. Coloured rectangles represent matches to binding sites: α‐box in blue, β‐box in green, E‐box in red, N‐box in purple and TATA‐box in black. The thick black line represents conserved fragments. Arrows indicate the transcription start site and ATG the beginning of the coding region. Note that the *sc SOPEs* are several kilobases upstream of the coding regions. *ase SOPE* from Gibert and Simpson (2003).

The *SOPE* is a regulatory element present in the *ac*,* sc* and *ase* genes of *D. melanogaster*. It is responsible for autoregulation and also mediates the lateral signalling that allows spacing of sensory organ precursors. *SOPEs* contain E‐boxes, binding sites for the proneural proteins themselves; N‐boxes, possible binding sites for Hairy/E(spl) proteins; α‐boxes, binding sites for NFκ‐B proteins; and β‐boxes, conserved sites for an unknown factor (Jarman *et al*., 1993; Ohsako *et al*., 1994; Van Doren *et al*., 1994; Culi & Modolell, 1998; Giagtzoglou *et al*., 2003; Ayyar *et al*., 2007). One blast2seq hit (40 105–40 129) corresponds to a 14‐bp fragment which is also the only stretch of sequence of the *sc SOPE* that is conserved in all *Drosophila* species sequenced (our observations). This 14‐bp fragment contains two adjacent binding sites, an E‐box and an N‐box separated by one nucleotide. We checked around this conserved sequence whether there were other α‐, β‐, E‐ or N‐boxes present in *C. vicina*. We found four E‐boxes, two α‐boxes and one β‐box. The organization of these binding sites in *C. vicina* is shown in Fig. 5, together with the SOPEs of *D. melanogaster*. Although individual and overlapping transcription factor binding sites are strongly conserved, there is no conservation of overall architecture (Fig. 5). A single N‐box is present in both species, but the number of E‐, α‐ and β‐boxes differs, as do their respective locations. The *C. vicina* sequence is about one and a half times bigger than that of *D. melanogaster*. Like that of *D. melanogaster*, the *C. vicina sc SOPE* is located some distance upstream of the *sc* coding sequence.

As these two enhancers (*L3/TSM* and *sc SOPE*) maintain their relative positions, we decided to check for the presence of the *ac SOPE*. In *D. melanogaster*, the *ac SOPE* is located outside the UTR but close to the transcription start site of *ac*. Although there are no blast2sequence hits in this region, we checked for the presence of binding sites in the first kilobase upstream of the *ac* coding region of *C. vicina*. We found three E‐boxes, one N‐box, two β‐boxes and one α‐box. The *D. melanogaster ac SOPE* is devoid of α‐boxes, but displays binding sites for Ac, an N‐box that is bound by Hairy (Van Doren *et al*., 1994) and a β‐box. Again, the SOPE of *ac* has conserved the location between *D. melanogaster* and *C. vicina* despite little conservation of its architecture (Fig. 5).

## Discussion

### Conservation of AS‐C architecture in *Calliphora vicina*

In our screen, we recovered *ac*, the only *ac*‐*sc* gene that had not been previously found in *C. vicina* (Figs 1 and S1). *ac* and *sc* are the most recent of the *ac‐sc* genes. They originated in the latest duplication, which occurred at the beginning of the diversification of the Cyclorrapha (Campuzano *et al*., 1985; Benos *et al*., 2001; Negre & Simpson, 2009). However, whereas *sc* displays 54% identity, similar to that found in *l'sc* (58%) or *ase* (53%), *ac* has only 33% identity at the protein level between *C. vicina* and *D. melanogaster*. The *ac* gene is also the only one not detected in the blast2sequence comparison. Thus, the earlier failure to clone *ac* was probably due to the fact that it is the most divergent of the *ac*‐*sc* proteins in cyclorraphous Diptera.

The *ac‐sc* orthologues of *C. vicina* are clustered into a complex very similar to that of *D. melanogaster*. The AS‐C of *C. vicina* contains the genes *ac*,* sc* and *l'sc* in the same order and orientation as in *D. melanogaster*. We were unable to reach the genes surrounding the AS‐C or to clone the *ase* region which is probably not present in the BAC library. Although *ase* is likely to be associated with the complex, the fact that it is the only gene of the AS‐C whose regulatory sequences are entirely contained within the UTR (Jarman *et al*., 1993), makes it possible that it could have separated from the rest of the complex.

A nonrelated gene, *pepsinogen‐like* (*pcl*), is located between *l'sc* and *ase* in the AS‐C of all *Drosophila* species examined (Campuzano *et al*., 1985; Benos *et al*., 2001; Negre & Simpson, 2009). This gene does not seem to be present at this location in *C. vicina*. Even though we have not sequenced the region containing *C. vicina ase*, we have identified conserved blocks of noncoding sequence in *C. vicina* that belong to the region around *pcl* (Table 2). We hypothesize that a transposition event moved the *pcl* gene into the AS‐C of *Drosophila* after the separation of the *Drosophila* and *Calliphora* lineages. In the *Drosophila* genus, this region has suffered several rearrangements, including gene duplication and further transpositions (Negre & Simpson, 2009).

The *C. vicina* region sequenced in this study is much larger than our initial estimate of the size of the AS‐C. Moreover, a comparison of noncoding sequence reveals that we have not reached the ends of the complex. In most insects sequenced, *yellow* and *P450* are next to (or within) the AS‐C (Negre & Simpson, 2009). In *C. vicina*, we did screen the library with the *y* gene, but no clones were recovered. We were also unable to ascertain whether other flanking genes are conserved, as these have not been reached. The region sequenced corresponds to approximately 78% of the *C. vicina* AS‐C. As this region shows a high conservation of overall structure, we would expect that the 130 kb still missing would be around 26‐kb upstream of *ac* (and including the DC enhancer) and 104‐kb downstream of *l'sc* and including the *ase* gene.

### Evolution of enhancer sequences of the *Calliphora vicina* AS‐C

We found 28 short stretches of noncoding sequence conserved between the AS‐C of *C. vicina* and both *D. melanogaster* and *D. virilis*. They are furthermore colinear. We find that 22 of the 28 conserved sequence blocks in *C. vicina* correlate with regions of known enhancer activity in *D. melanogaster*. This therefore suggests that most of the conserved sequences are functional and could correspond to enhancers acting in a manner similar to those of *D. melanogaster*. The conservation and colinearity of these sequences suggests not only a conserved structure of the regulatory elements of the AS‐C in both species, but also a common origin. However, as the *ac* gene was not detected in the blast2sequence comparison, other homologous sequences have probably escaped detection.

Of the three well‐defined regulatory modules from *D. melanogaster* (the *DCE*, the *SOPE* and the *L3/TSM*), the *DCE* is unfortunately just outside the region sequenced. *SOPEs* are present in three AS‐C genes (*ac*,* sc* and *ase*). In *C. vicina*, we have identified the *ac SOPE* by its conserved location and the *sc SOPE* by a conserved block. The *ase SOPE* lies outside the sequenced region but had been described in a previous study (Gibert & Simpson, 2003). Finally, we have identified the *L3/TSM* enhancer by a conserved block. The comparison of *D. melanogaster* and *C. vicina L3/TSM* sequences, combined with new data about binding site composition of homeobox proteins, shows the presence and conservation of Ara binding sites and En/Antp binding sites in this enhancer. These sites seem to be the core of this enhancer, and other transcription factors have not been yet identified.

Previous comparison of the *SOPEs* between different arthropods reveals the presence of binding sites for the same factors, but no conservation of spatial architecture: the number of sites, their orientation and spacing differs between species. In contrast, within Diptera, the *ase SOPE* displays much greater conservation. Short tracts of sequence are highly conserved between drosophilids as well as with *Ceratitis capitata* and *C. vicina* (Gibert & Simpson, 2003). The conserved stretches contain binding sites for the known transcriptional regulators. Remarkably, the number and ordering of sites is conserved between *D. melanogaster*,* C. capitata* and *C. vicina* even though the size of the enhancer has changed (Fig. 5) (Gibert & Simpson, 2003) (our observations).

Here, we describe the *SOPEs* of *ac* and *sc* of *C. vicina*. They appear to have diverged from those of *D. melanogaster* to a greater extent than the *ase SOPE*, because neither the number of binding sites nor their order is conserved. Why have they evolved so differently? One possibility is that the *ase SOPE* is constrained by mRNA folding because of its location in the UTR. Other explanations might reside in the different mode of functioning between *ase* and *ac‐sc*. Unlike *ase*, which is exclusively regulated by the *SOPE*,* ac* and *sc* are regulated by a number of different *cis‐*regulatory elements, each of which presumably needs to loop up in proximity to the promoter. They are first expressed in proneural stripes/clusters and then restricted to developing neural precursors. The *ac* and *sc* proteins are structurally very similar, are co‐expressed during neural development and furthermore have been shown to cross activate one another within neural precursors (Martinez & Modolell, 1991; Gomez‐Skarmeta *et al*., 1995). The two genes probably act in a redundant fashion to drive neural development. In fact, *ac* has been shown to be dispensable in *D. melanogaster* (Marcellini *et al*., 2005). In contrast, *ase* is activated by high levels of both Ac and Sc and its expression is restricted to neural precursors.

The genes of the AS‐C originated by duplication from an *ASH/ase* ancestor. The sequence signatures and conserved position of the *ase* SOPE enhancers suggest that an SOPE enhancer was already present in the UTR of the *ASH*/*ase* ancestor prior to the *ASH/ase* split (Ayyar *et al*., 2010). Its position in the UTR is thought to be the ancestral location of this element. It is likely that after subsequent gene duplications, the SOPE was duplicated along with the coding sequences. The *ase SOPE* has been retained in the UTR of *ase* in arthropods. The *ASH* SOPE appears to have moved outside the UTR and evolved differently in different lineages/genes. Within the Diptera, the SOPE remained associated with the *ac*/*sc* homologue after the duplication that gave rise to *l'sc*, but appears to have been lost from the *l'sc* gene. It was probably further duplicated at the origin of the *ac* and *sc* genes but has become separated from the transcription unit in *ac* and *sc* of both *D. melanogaster* and *C. vicina*, presumably after duplication of the ancestral proneural *ac‐sc* precursor gene (Ayyar *et al*., 2010). Although it stayed close to the UTR in *ac*, it moved several kilobases upstream in the *sc* homologues.

Most enhancers of specific genes have a common origin and are bound by the same transcription factors. Their sequences, however, turn over rapidly and cannot generally be aligned having evolved compensatory mutations to maintain the degree of binding required. blast is able to detect short conserved sequences (e.g. containing one or two binding sites) independently of their orientation. A comparison of the *even‐skipped* enhancers between *Drosophila* and sepsids revealed that, even though they are highly diverged, one or more small nearly identical sequence blocks could be identified within each enhancer (Hare *et al*., 2008). The blocks were found to be enriched in known binding sites, especially paired ones. We find a similar pattern of conservation within the *sc SOPE* and the *L3/TSM* enhancers: an enrichment of adjacent and unique sites within small islands of strong sequence conservation. This has generally been considered the result of purifying selection and an indicator of the functional importance of these configurations for proper enhancer function, although Lusk and Eisen (Lusk & Eisen, 2010) have shown that this clustering of sites could also result from selection for binding site composition alone together with the bias in *D. melanogaster* for deletions over insertions.

The sequence comparison we present has allowed us to identify the core elements of some of the enhancers in the AS‐C of *C. vicina*. The total length of sequence required for function of each enhancer has not been determined and remains a challenge. We find overall conserved synteny along the AS‐C gene complex. Conservation of enhancer order (with minimal intralocus inversions) is also observed in the *even‐skipped* locus in *Drosophila*, Sepsids, Tephritids and in the Hox genes in Drosophilids (Negre *et al*., 2005; Hare *et al*., 2008; Peterson *et al*., 2009). Only minor rearrangements changing the orientation of small fragments were detected in our study. Such a lack of rearrangements is consistent with the fact that enhancers functioning to drive expression in different tissues sometimes overlap and with the existence of regulatory elements driving expression from one or more coding regions that need to be in close proximity, as previously shown in *D. melanogaster* (Ruiz‐Gomez & Modolell, 1987; Ghysen & Dambly‐Chaudiere, 1988). There is no evidence to suggest that the spatial arrangement of the regulatory elements is important for their function.

## Conclusions

We propose that the organization of regulatory sequences, like that of coding sequences, is conserved between *D. melanogaster* and *C. vicina* despite a divergence time of 150 Myr. Synteny is conserved not only for coding sequences but also for stretches of noncoding sequences, some of which correspond to known enhancers. This overall conservation of the architecture of regulatory elements implies a common evolutionary origin for the regulatory modules. If so, the expression patterns might also predate the divergence between these two species. This would be consistent with the hypothesis that, for example, the diverse bristle arrangements of different species are derived from a common underlying ancestral pattern (Simpson *et al*., 1999; Pistillo *et al*., 2002). For the *SOPEs*, we suggest that, like their associated genes, they date back to the *Drosophila*/*Calliphora* ancestor and probably originated by duplication along with their target genes. Duplication and subfunctionalization could be a common source of regulatory elements as happens with coding genes. To determine whether other regulatory elements arose from duplication and subsequent divergence along with the duplication of coding sequences, an examination of the AS‐C of species indicative of the state of the complex prior to some or all of the duplication events is required.

## Supporting information


**Figure S1** Aminoacid alignment of the *ac* protein in Diptera.

**Figure S2** Non‐coding sequence alignments in the AS‐C between *Drosophila melanogaster* and *Calliphora vicina*.

**Table S1** Conserved blocks detected with mVISTA.
