# Supplementary material for: The achaete‐scute complex in Diptera: patterns of noncoding sequence evolution
Source: J Evol Biol. 2015 Sep 7;28(10):1770–81. doi: 10.1111/jeb.12687 (PMC4832353; doi:10.1111/jeb.12687)
Supplement: Supplementary file 1 — Figure S1 Aminoacid alignment of the ac protein in Diptera. [file JEB-28-1770-s001.doc]

BAD AVG GOOD

Cvic_ac   :  73

Dmel_ac   :  73

cons      :  73

Cvic_ac   MALGSENMVYHQQPTQYQLQQHHAQQQPLREVLRTRNVNHQRSIAPAPYTKPSAHNSMLAGTTLDSNNPAVVKRNARERSRVKQVNDGFSTLRKHIPTAI

Dmel_ac   MALGSENHSVFNDDE---------------------------------------------ESSSAFNGPSVIRRNARERNRVKQVNNGFSQLRQHIPAAV

      *******   .::                                                ::   *.*:*::******.******:*** **:***:*:

Cvic_ac   VAEISNGRRGIGPGADKKLSKVDTLRMAAEYIRRLKNLIDEVDSSDSSSVSSYGTTTNSPVSSYASSTSSSPPPSLYINNTTTSGNNYYQQSQAQTSLQY

Dmel_ac   IADLSNGRRGIGPGANKKLSKVSTLKMAVEYIRRLQKVLHENDQQKQKQLHLQ------------------------------QQHLHFQQQQQHQHL--

      :*::***********:******.**:**.******::::.* *......:                                 . : ::**.* :  *

Cvic_ac   CTPLSYAGSFQQHNLISPANSCTSHESYQFHHSPNHYQSSDPSTFALNTPNTTPIKTEPTSSSFSFDDYQHNNTSSASSTDDEELLDYISLWQD-Q

Dmel_ac   -----YA-WHQELQLQSPTGSTSSCNSISSYC--------KPATS--TIPGATPPNNFHTKLEASFEDYRNNS--CSSGTEDEDILDYISLWQDDL

           **  .*: :* **:.* :* :* . :         .*:*   . *.:** :.  *. . **:**::*.  .:*.*:**::*********

BAD AVG GOOD

Cvic_ac   :  74
Mdom_ac   :  75
Dmel_ac   :  76
Dvir_ac   :  74
cons      :  75

Cvic_ac   MALGSENMVYHQQPTQY----QLQQHHAQQQPLREVLRTRNVNHQRSI-APAPYTKPSAHNSM---------LAGTTLDSNNPAVVKRNARERSRVKQVN
Mdom_ac   MALGSENMMYHHQSATTVTKFQYQK-QVLHTPPREVLRTRNVN-QRSI-APAPYNKPATKS------------EGTTLDSNNPSVIRRNARERNRVKQVN
Dmel_ac   MALGSENHSVF-----------------------------------------------------------NDDEESSSAFNGPSVIRRNARERNRVKQVN
Dvir_ac   MALGSENQSRS------------------------------------LYRLAPYAKPSSNNYLQQQQQLLLSEQQQPMLLGGQSVVRRNARERNRVKQVN
       *******                                                                     .   .. :*::******.******

Cvic_ac   DGFSTLRKHIPTAIVAEISNGRRGIGPGADKKLSKVDTLRMAAEYIRRLKNLIDEVDSSDS-SSVSSYGTTTNSPVSSYASSTSSSPPPS-LYINNTT--
Mdom_ac   DGFTHLRQHIPTAIIAEISNGRRGIGPGADKKLSKVDTLRMAAEYIRRLKKLIDDVDSGSDSSSVSSYGSLSAASPSYSSSSNSGSPPPAPLHQQQTSNA
Dmel_ac   NGFSQLRQHIPAAVIADLSNGRRGIGPGANKKLSKVSTLKMAVEYIRRLQKVLHENDQQKQ-KQL---------------------------HL------
Dvir_ac   NGFSQLRQHIPVAIIADLSNGRRGIGPGANKKLSKVSTLRMAVEYIRRLQRLIDDNDQQQQ-QQQQ---------------QQLLS-PQQQ-LA------
       :**: **:***.*::*::***********:******.**:**.******:.::.: *. .. ..                                    

Cvic_ac   -TSGNNYYQQSQAQTSLQYCTPLSYAGSFQQHNLISPANSCTSH----ESYQFHHSPNHYQSSDPSTFALNTPNTTPIKTEPTSSSFSFDDYQHNNTSSA
Mdom_ac   PQLNNMYYQQQTQFYTQPHHPSLSYAGSFQQHNLISPANSTSSSTYSAELYPQQESPQHYQSSGFEYANNITPATTPIKFEP----VSFEDYNHNSNSS-
Dmel_ac   -QQQHLHFQQQQ-------QHQHLYA-WHQELQLQSPTGSTSSCN-SISSYCKPATST-------------IPGATPPNNFHTKLEASFEDYRNNSCS--
Dvir_ac   -QQQQFSYQQQQLQQ----QQQQFYA-LQQQLQLISPGGSSCSSNASNDSNASNSSSS------------NYYATQPTELKLESSGGSYEDYPNNSCS--
           :  :**.             **   *: :* ** .*  *     .      :.                 : * :        *::** :*. *  

Cvic_ac   SSTDDEELLDYISLWQD-Q
Mdom_ac   SSAEDEELLDYISLWQD-E
Dmel_ac   SGTEDEDILDYISLWQDDL
Dvir_ac   SGAEDDDILDYISLWQDDI
       *.::*:::*********  
