# Supplementary material for: The achaete‐scute complex in Diptera: patterns of noncoding sequence evolution
Source: J Evol Biol. 2015 Sep 7;28(10):1770–81. doi: 10.1111/jeb.12687 (PMC4832353; doi:10.1111/jeb.12687)
Supplement: Supplementary file 2 — Figure S2 Non‐coding sequence alignments in the AS‐C between Drosophila melanogaster and Calliphora vicina. [file JEB-28-1770-s002.pdf]

Supplementary Figure 2. Non-coding sequence alignments in the AS-C between *D. melanogaster* and *C. vicina*. Sequences alignments and scores of the blast2sequence hits summarized in Table 2. Hits are ordered by BAC sequence. Binding sites are highlighted in bold and the transcription factor is indicated underneath.

```
Query=  Dmel_CrX_210000_330000
Length=120001

>lcl|36649 113H10
Length=96426

 Score = 37.4 bits (40),  Expect = 0.066
 Identities = 41/57 (71%), Gaps = 16/57 (28%)
 Strand=Plus/Plus

Query  13423  CATGCAAATAATATAAGTTACTGTGTCAGCGACAAAAGGCAACAGGTGAAATTGTCG  13479
              ||||||||||   |||||||||||||             ||||||||||||||||||
Sbjct  18171  CATGCAAATA---TAAGTTACTGTGT-------------CAACAGGTGAAATTGTCG  18211


 Score = 44.6 bits (48),  Expect = 4e-04
 Identities = 61/84 (72%), Gaps = 10/84 (11%)
 Strand=Plus/Plus

Query  14226  CGTGAGTGCCAGAAGCTGATTTCAGCGCGGCTACCGATAAG-----CAACCGCTTCCAGG  14280
              |||||||||||||||||  |||      |||||||||||||      ||| || | | ||
Sbjct  20773  CGTGAGTGCCAGAAGCTTTTTT----TGGGCTACCGATAAGGCAGCTAACTGC-TACGGG  20827

Query  14281  TTTGACAGACGCGTTGATTGGTTT  14304
                ||||||   |||||||||||||
Sbjct  20828  AGTGACAGGTCCGTTGATTGGTTT  20851


 Score = 39.2 bits (42),  Expect = 0.019
 Identities = 24/26 (92%), Gaps = 0/26 (0%)
 Strand=Plus/Minus

Query  15660  AAAAAATAATAATAAAATTGCAAATT  15685
              |||||||||||| |||||| ||||||
Sbjct  39844  AAAAAATAATAAAAAAAATTACAAATT  39819


 Score = 37.4 bits (40),  Expect = 0.066
 Identities = 22/23 (95%), Gaps = 0/23 (0%)
 Strand=Plus/Minus

Query  20754  AAAATATGGGTTATATATTTAAA  20776
              |||||||||||||| ||||||||
Sbjct  51870  AAAATATGGGTTATTTATTTAAA  51848


 Score = 46.4 bits (50),  Expect = 1e-04
 Identities = 31/35 (88%), Gaps = 0/35 (0%)
 Strand=Plus/Plus

Query  27904  TTTAGAAAATGTTTAATAGTaaaaaaaaaTTTATT  27938
              ||||||||||||||||||||||| | |  ||||||
Sbjct  95378  TTTAGAAAATGTTTAATAGTAAATATATGTTTATT  95412



 Score = 37.4 bits (40),  Expect = 0.066
 Identities = 33/41 (80%), Gaps = 3/41 (7%)
 Strand=Plus/Plus
```

```
Query  29125  TTAAATTTATATGTACA---TAATGTTTAATGTTTTATTTT  29162
              ||| ||  ||||||| |   ||||||||||||||||| ||
Sbjct  52401  TTATATACATATGTATAATGTAATGTTTAATGTTTTATATT  52441


 Score = 41.0 bits (44),  Expect = 0.005
 Identities = 25/27 (92%), Gaps = 0/27 (0%)
 Strand=Plus/Minus

Query  47873  AAATTTATTTAAtttttttCAATAAA  47899
              ||||||||||| ||||||||||| |||
Sbjct  21976  AAATTTATTTATTTTTTTTCAAGAAA  21950


 Score = 37.4 bits (40),  Expect = 0.066
 Identities = 25/28 (89%), Gaps = 0/28 (0%)
 Strand=Plus/Plus

Query  55566  AATTAATTGAATTCAGTTTTTGTTTGTT  55593
              ||| |||||||||||||||| ||| ||
Sbjct  4144   AATAAATTGAATTCAGTTTTTTTTTATT  4171


>lcl|18255 CV99M22
Length=102758

 Score = 96.9 bits (106),  Expect = 9e-20
 Identities = 59/63 (93%), Gaps = 0/63 (0%)
 Strand=Plus/Minus

Query  19125  TGCGACAGCAAACGGTGCAAAATCGAACGCTTTTTGTGTGAAGTTAATAATACGTTTGAC  19184
              |||||||||||||||||||||| || |||||||||||||||| ||||||||||||||| |
Sbjct  40733  TGCGACAGCAAACGGTGCAAAAACGGACGCTTTTTGTGTGAATTTAATAATACGTTTGTC  40674

Query  19185  ATG  19187
              |||
Sbjct  40673  ATG  40671


 Score = 46.4 bits (50),  Expect = 1e-04
 Identities = 30/33 (90%), Gaps = 0/33 (0%)
 Strand=Plus/Minus

Query  19665  ATTTATGATTTATTAAGGATATTCTACTTACAA  19697
              |||||||||||||||||||| ||||  ||||||
Sbjct  37506  ATTTATGATTTATTAAGGATGTTCTGATTACAA  37474


 Score = 46.4 bits (50),  Expect = 1e-04
 Identities = 28/30 (93%), Gaps = 0/30 (0%)
 Strand=Plus/Plus

Query  23323  AAGGTGCTAAATGGGGCGTACTAATGGGAT  23352
              |||||| ||||||||||||||||||| |||
Sbjct  57812  AAGGTGTTAAATGGGGCGTACTAATGAGAT  57841


 Score = 66.2 bits (72),  Expect = 1e-10
 Identities = 36/36 (100%), Gaps = 0/36 (0%)
 Strand=Plus/Minus

Query  25810  ATAATAAATTGAATGACATTCATAAATAAACAATAT  25845
              ||||||||||||||||||||||||||||||||||||
Sbjct  73435  ATAATAAATTGAATGACATTCATAAATAAACAATAT  73400
```

```
 Score = 37.4 bits (40),  Expect = 0.070
 Identities = 25/28 (89%), Gaps = 0/28 (0%)
 Strand=Plus/Plus

Query  29758  ATTCCATTGGTACATAAATGACATTAAA  29785
              |||||| |||  |||||||||||||||
Sbjct  38755  ATTCCACTGGCCCATAAATGACATTAAA  38782


 Score = 37.4 bits (40),  Expect = 0.070
 Identities = 22/23 (95%), Gaps = 0/23 (0%)
 Strand=Plus/Minus

Query  54261  GTAAGAATACAGCAAATAAATAA  54283
              ||||||||||||||| |||||||
Sbjct  37751  GTAAGAATACAGCAACTAAATAA  37729


>lcl|1257 CV97L04
Length=111044

 Score = 55.4 bits (60),  Expect = 3e-07
 Identities = 38/42 (90%), Gaps = 1/42 (2%)
 Strand=Plus/Plus

Query  33788  GGCGATGAACGAATTTATTGCAACAACGC-ACACTATAATTG  33828
              |||||| ||||||||||||||||||||||| | || |||||||
Sbjct  44884  GGCGATAAACGAATTTATTGCAACAACGCTAAACAATAATTG  44925


 Score = 60.8 bits (66),  Expect = 7e-09
 Identities = 39/43 (90%), Gaps = 0/43 (0%)
 Strand=Plus/Minus

Query  34886  AGGCTGACACTTTAATTTTCAATTACAACAAATTGCCTATAAT  34928
              ||| ||||||||| ||||||||||||||||||||||  |||||
Sbjct  50672  AGGGTGACACTTTGATTTTCAATTACAACAAATTGCTCATAAT  50630


 Score = 44.6 bits (48),  Expect = 5e-04
 Identities = 26/27 (96%), Gaps = 0/27 (0%)
 Strand=Plus/Plus

Query  37687  ATTTATAATGTGTGGTCGTTGAATAAT  37713
              |||||| ||||||||||||||||||||
Sbjct  24868  ATTTATTATGTGTGGTCGTTGAATAAT  24894


 Score = 41.0 bits (44),  Expect = 0.006
 Identities = 24/25 (96%), Gaps = 0/25 (0%)
 Strand=Plus/Minus

Query  38125  TGATTAATGAGTTTTTAAGAGGCTC  38149
              |||||||||| ||||||||||||||
Sbjct  70125  TGATTAATGATTTTTTAAGAGGCTC  70101


 Score = 37.4 bits (40),  Expect = 0.076
 Identities = 23/25 (92%), Gaps = 0/25 (0%)
 Strand=Plus/Minus

Query  40105  TAAATACACCTGCCACGCGTCGCCA  40129
              ||||||||||||||||||| || ||
Sbjct  90093  TAAATACACCTGCCACGCGCCGTCA  90069
                    E-box  N-box
```

```
 Score = 64.4 bits (70),  Expect = 5e-10
 Identities = 43/48 (89%), Gaps = 0/48 (0%)
 Strand=Plus/Plus

Query  42356  TTTGATAATTTAATTGGAGAAATAAGTGAAATTGTTTGAACACCTTTA  42403
              ||||||  ||||||| ||||||||| ||||||||||||||||||| ||
Sbjct  99269  TTTGATCGTTTAATTAGAGAAATAATTGAAATTGTTTGAACACCTATA  99316
                       En/Antp            Ara/Caup  Ara/Caup


 Score = 66.2 bits (72),  Expect = 2e-10
 Identities = 54/66 (81%), Gaps = 0/66 (0%)
 Strand=Plus/Plus

Query  42858   GTACATAACGCGAGGGTTTAGGACGAAGGGACTCATTCTTGTGTAAGGTGTCAAACGATC  42917
               |||| ||| || | |||||||||| |||||||||||||||    ||||||||  | ||||
Sbjct  104855  GTACTTAATGCAAAGGTTTAGGACAAAGGGACTCATTCTTAATCAAGGTGTCGTAGGATC  104914

Query  42918   AAGTTC  42923
               ||||||
Sbjct  104915  AAGTTC  104920


 Score = 37.4 bits (40),  Expect = 0.076
 Identities = 26/30 (86%), Gaps = 0/30 (0%)
 Strand=Plus/Minus

Query  109968  TTGAATAAATGCAAATAAATATGAACATTT  109997
               || |||||||  ||||| ||||||||||||
Sbjct  107083  TTTAATAAATTTAAATATATATGAACATTT  107054


>lcl|60883 CV62B24
Length=90178

 Score = 37.4 bits (40),  Expect = 0.062
 Identities = 23/25 (92%), Gaps = 0/25 (0%)
 Strand=Plus/Minus

Query  40105  TAAATACACCTGCCACGCGTCGCCA  40129
              ||||||||||||||||||| || ||
Sbjct  14103  TAAATACACCTGCCACGCGCCGTCA  14079
                    E-box  N-box

 Score = 64.4 bits (70),  Expect = 4e-10
 Identities = 43/48 (89%), Gaps = 0/48 (0%)
 Strand=Plus/Plus

Query  42356  TTTGATAATTTAATTGGAGAAATAAGTGAAATTGTTTGAACACCTTTA  42403
              ||||||  ||||||| ||||||||| ||||||||||||||||||| ||
Sbjct  21655  TTTGATCGTTTAATTAGAGAAATAATTGAAATTGTTTGAACACCTATA  21702
                       En/Antp            Ara/Caup  Ara/Caup


 Score = 66.2 bits (72),  Expect = 1e-10
 Identities = 54/66 (81%), Gaps = 0/66 (0%)
 Strand=Plus/Plus

Query  42858  GTACATAACGCGAGGGTTTAGGACGAAGGGACTCATTCTTGTGTAAGGTGTCAAACGATC  42917
              |||| ||| || | |||||||||| |||||||||||||||    ||||||||  | ||||
Sbjct  27740  GTACTTAATGCAAAGGTTTAGGACAAAGGGACTCATTCTTAATCAAGGTGTCGTAGGATC  27799

Query  42918  AAGTTC  42923
              ||||||
Sbjct  27800  AAGTTC  27805
```

```
 Score = 44.6 bits (48),  Expect = 4e-04
 Identities = 29/32 (90%), Gaps = 0/32 (0%)
 Strand=Plus/Minus

Query  46110  GGCGTTATTGATTAGCGTTATGAATTATTGAT  46141
              ||||||||  |||||| |||||||||||||||
Sbjct  47973  GGCGTTATCAATTAGCTTTATGAATTATTGAT  47942


 Score = 46.4 bits (50),  Expect = 1e-04
 Identities = 32/35 (91%), Gaps = 1/35 (2%)
 Strand=Plus/Minus

Query  46525  TGATAAATGTAGTTACATACCAA-ATGTGATAACT  46558
              |||||||||||||||||||| || | |||||||||
Sbjct  46965  TGATAAATGTAGTTACATACAAATAAGTGATAACT  46931


 Score = 59.0 bits (64),  Expect = 2e-08
 Identities = 41/47 (87%), Gaps = 0/47 (0%)
 Strand=Plus/Minus

Query  51057  GGTAATATGGTTCAACACCAATTTACACACATCATGCATCTTCTTAG  51103
              |||||||| |||||||||||||||| |||| || | |||||| ||||
Sbjct  72470  GGTAATATTGTTCAACACCAATTTAGACACTTCGTTCATCTTGTTAG  72424


 Score = 50.0 bits (54),  Expect = 1e-05
 Identities = 30/32 (93%), Gaps = 0/32 (0%)
 Strand=Plus/Minus

Query  53731  TAAATTACCGTTATTAGGTGTGATCGTGCGAA  53762
              ||||| ||||||||||||||||||||||| ||
Sbjct  89286  TAAATAACCGTTATTAGGTGTGATCGTGCCAA  89255


 Score = 37.4 bits (40),  Expect = 0.062
 Identities = 29/34 (85%), Gaps = 2/34 (5%)
 Strand=Plus/Minus

Query  93603  ACAATGTGATAT--AATAACTTCATTTAAATAAA  93634
              |||| || ||||  |||||||||||| |||||||
Sbjct  29823  ACAAAGTTATATTAAATAACTTCATTAAAATAAA  29790


>lcl|54387 CV16B10
Length=135393

 Score = 37.4 bits (40),  Expect = 0.092
 Identities = 25/27 (92%), Gaps = 1/27 (3%)
 Strand=Plus/Plus

Query  32258   TTTAAAATATTTTTAAAAAATCTGTAA  32284
               ||||||||||||||  |||||||||||
Sbjct  118674  TTTAAAATATTTTT-CAAAATCTGTAA  118699


 Score = 59.0 bits (64),  Expect = 3e-08
 Identities = 41/47 (87%), Gaps = 0/47 (0%)
 Strand=Plus/Minus

Query  51057  GGTAATATGGTTCAACACCAATTTACACACATCATGCATCTTCTTAG  51103
              |||||||| |||||||||||||||| |||| || | |||||| ||||
Sbjct  2419   GGTAATATTGTTCAACACCAATTTAGACACTTCGTTCATCTTGTTAG  2373
```

```
 Score = 57.2 bits (62),  Expect = 1e-07
 Identities = 43/50 (86%), Gaps = 2/50 (4%)
 Strand=Plus/Minus

Query  51964  TACTTTTAATGATAATTGCCGTCTCACAACTCATTTTTCACAATGATAAA  52013
              || ||||||||||||||||  |   |||||||||||||||| |||||||
Sbjct  30646  TAATTTTAATGATAATTGC--TACAACAACTCATTTTTCACATTGATAAA  30599


 Score = 87.8 bits (96),  Expect = 6e-17
 Identities = 78/94 (82%), Gaps = 4/94 (4%)
 Strand=Plus/Minus

Query  52755  AAACTTATTTCGCTGTTTTT-GCAATTTAAGGTAATTATTGTGTACCTTTGGGGTAATTT  52813
              ||| |||||||  || |||  |||||||||||||||||  |||  ||  ||||||||||
Sbjct  24209  AAATTTATTTCTTTGATTTCAGCAATTTAAGGTAATTATAATGTTGCTAAGGGGTAATTT  24150

Query  52814  TAACACCTCGACACC--ATCG-ACAAATATCATC  52844
              |||||| ||||||||  |||| ||||||||||||
Sbjct  24149  TAACACTTCGACACCAAATCGTACAAATATCATC  24116


 Score = 50.0 bits (54),  Expect = 1e-05
 Identities = 30/32 (93%), Gaps = 0/32 (0%)
 Strand=Plus/Minus

Query  53731  TAAATTACCGTTATTAGGTGTGATCGTGCGAA  53762
              ||||| ||||||||||||||||||||||| ||
Sbjct  19235  TAAATAACCGTTATTAGGTGTGATCGTGCCAA  19204


 Score = 44.6 bits (48),  Expect = 6e-04
 Identities = 85/121 (70%), Gaps = 7/121 (5%)
 Strand=Plus/Plus

Query  57555  tttttCATAGTTATAAGTTTGTTATA---AGCATGGAAGACACTAAACTAACTACTT-TT  57610
              |||||  || || ||||||| ||||    |  ||||||||||||||||  ||| || ||
Sbjct  50662  TTTTTATTAATTTTAAGTTTATTATTTTTAATATGGAAGACACTAAACT--CTAGTTATT  50719

Query  57611  AAGCCAAAATAAAAACATATTGATAAATTTAATTCCAAATGTTTTTTACTGAAATCACTT  57670
              |||| |||  | ||||   || | |||  |  ||  |||| |||||||   |||| | ||
Sbjct  50720  AAGCAAAACAACAAACCCCTTTAAAAAAATTGTTATAAAT-TTTTTATATAAATTATTT  50778

Query  57671  A  57671
              |
Sbjct  50779  A  50779


 Score = 37.4 bits (40),  Expect = 0.092
 Identities = 36/45 (80%), Gaps = 1/45 (2%)
 Strand=Plus/Plus

Query  60987  AATCGATTTATTAAAATAAAAAGCAAGGCAAAACAAAAGATTGTC  61031
              |||||||||||| | || |||| ||| | |||||| || | ||||
Sbjct  70885  AATCGATTTATTTATATGAAAATCAA-GTAAAACATAAAAGTGTC  70928


 Score = 39.2 bits (42),  Expect = 0.026
 Identities = 24/26 (92%), Gaps = 0/26 (0%)
 Strand=Plus/Minus

Query  66739   TGTTTGGCAATTAATTAAATTAAGTT  66764
               ||||| |||||||||||||||| ||
Sbjct  117858  TGTTTTGCAATTAATTAAATTAAATT  117833
                         Homeodomain
```

(In the last alignment, TTAATTAA is printed in bold in both Query and Sbjct.)

```
>lcl|6909 104L14
Length=115595

 Score = 37.4 bits (40),  Expect = 0.079
 Identities = 31/38 (81%), Gaps = 0/38 (0%)
 Strand=Plus/Plus

Query  41089  TTTATGAAATATAAAATTTAAATCATTCAAAATTAAAA  41126
              ||||||||||| | ||||| | || ||| || ||||||
Sbjct  67809  TTTATGAAATACACAATTTTATTCGTTCGAATTTAAAA  67846


 Score = 42.8 bits (46),  Expect = 0.002
 Identities = 28/31 (90%), Gaps = 0/31 (0%)
 Strand=Plus/Plus

Query  68213  GCAATCAAAAATCAATTCATAAACAATTGCA  68243
              ||||||||||| ||||||||||| | |||||
Sbjct  28982  GCAATCAAAAAACAATTCATAAAAATTTGCA  29012


 Score = 39.2 bits (42),  Expect = 0.023
 Identities = 24/26 (92%), Gaps = 0/26 (0%)
 Strand=Plus/Plus

Query  68286  AATCAAATTTGTTATAAAACTGTGAA  68311
              || |||||||||||||||||||| ||
Sbjct  29081  AAACAAATTTGTTATAAAACTGTCAA  29106


 Score = 39.2 bits (42),  Expect = 0.023
 Identities = 23/24 (95%), Gaps = 0/24 (0%)
 Strand=Plus/Plus

Query  69400  GTATGAAGCGATCAATCAAAAATG  69423
              |||| |||||||||||||||||||
Sbjct  32865  GTATAAAGCGATCAATCAAAAATG  32888


 Score = 51.8 bits (56),  Expect = 4e-06
 Identities = 43/50 (86%), Gaps = 2/50 (4%)
 Strand=Plus/Plus

Query  69638  TGATGTACAGTTTGTTAGAATTTGAATTGTTCGGAG-CGCATCGTAAATT  69686
              |||| ||||||||||||||||||||||| |    || |||||||||||||
Sbjct  32987  TGATATACAGTTTGTTAGAATTTGAATT-TAAATAGACGCATCGTAAATT  33035


 Score = 46.4 bits (50),  Expect = 2e-04
 Identities = 25/25 (100%), Gaps = 0/25 (0%)
 Strand=Plus/Minus

Query  72901  ATCTGATTAAGAATATTTCAATTAG  72925
              |||||||||||||||||||||||||
Sbjct  26919  ATCTGATTAAGAATATTTCAATTAG  26895


 Score = 69.8 bits (76),  Expect = 1e-11
 Identities = 49/56 (87%), Gaps = 0/56 (0%)
 Strand=Plus/Plus

Query  86922  GTGAATTTATTATGTGATTTTGATCATTTGACATAATCTATTTAGCGTCTGCTCTC  86977
              || ||||||| |||||||| ||||| ||| ||||||||||||||||  ||||||||
Sbjct  54520  GTAAATTTATGATGTGATTTCGATCAATTGGCATAATCTATTTAGCGCATGCTCTC  54575
```

```
 Score = 37.4 bits (40),  Expect = 0.079
 Identities = 25/27 (92%), Gaps = 2/27 (7%)
 Strand=Plus/Plus

Query  89432  TTCAATT--AAATTGACAAATTTTTAT  89456
              |||||||  ||||||||||||||||||
Sbjct  55287  TTCAATTTTAAATTGACAAATTTTTAT  55313


 Score = 51.8 bits (56),  Expect = 4e-06
 Identities = 28/28 (100%), Gaps = 0/28 (0%)
 Strand=Plus/Plus

Query  90692  AATATTTAACTTTGAACTAATTATTTCA  90719
              ||||||||||||||||||||||||||||
Sbjct  69009  AATATTTAACTTTGAACTAATTATTTCA  69036


 Score = 42.8 bits (46),  Expect = 0.002
 Identities = 35/43 (81%), Gaps = 0/43 (0%)
 Strand=Plus/Minus

Query  96409  TTTCAATTCCCGATGGTCCAATTCAAGTTCACCACTTGCACTT  96451
              ||| | ||||| | |||  ||| |||||||| |||||||||||
Sbjct  90974  TTTAATTTCCCCAGGGTGTAATACAAGTTCAACACTTGCACTT  90932
                                              E-box
```
