# Supplementary material for: The achaete‐scute complex in Diptera: patterns of noncoding sequence evolution
Source: J Evol Biol. 2015 Sep 7;28(10):1770–81. doi: 10.1111/jeb.12687 (PMC4832353; doi:10.1111/jeb.12687)
Supplement: Supplementary file 3 — Table S1 Conserved blocks detected with mVISTA. [file JEB-28-1770-s003.doc]

mVISTA

AVID

Calc Window, bp:

100

Min Cons Width, bp:

25

Cons Identity, %:

85

Minimum Y, %:

30

***** Conserved Regions - sequence1 Dmel_CrX_210000_330000 (sequence2 113H10) *****

10965 (16392) to 10989 (16417) = [26bp](http://pipeline.lbl.gov/cgi-bin/gp_cns?server=localhost&db=gp_server&run=u99-GXiIubQd&base=338&pos=Dmel_CrX_210000_330000:10965-10989&align_id=6&org=56&length=26&identity=88.5&type=intergenic&output=mfa) at 88.5% intergenic

13423 (18171) to 13450 (18195) = [28bp](http://pipeline.lbl.gov/cgi-bin/gp_cns?server=localhost&db=gp_server&run=u99-GXiIubQd&base=338&pos=Dmel_CrX_210000_330000:13423-13450&align_id=6&org=56&length=28&identity=89.3&type=intergenic&output=mfa) at 89.3% intergenic

Total [54bp](http://pipeline.lbl.gov/cgi-bin/gp_cns?server=localhost&db=gp_server&run=u99-GXiIubQd&base=338&pos=Dmel_CrX_210000_330000:1-31433&align_id=6&org=56&genes=user&min_id=85&min_length=25&output=cns_mfa) at 88.9%

***** Conserved Regions - sequence1 Dmel_CrX_210000_330000 (sequence3 CV99M22) *****

29758 (38755) to 29785 (38782) = [28bp](http://pipeline.lbl.gov/cgi-bin/gp_cns?server=localhost&db=gp_server&run=u99-GXiIubQd&base=338&pos=Dmel_CrX_210000_330000:29758-29785&align_id=7&org=57&length=28&identity=89.3&type=intergenic&output=mfa) at 89.3% intergenic

Total [28bp](http://pipeline.lbl.gov/cgi-bin/gp_cns?server=localhost&db=gp_server&run=u99-GXiIubQd&base=338&pos=Dmel_CrX_210000_330000:1-31433&align_id=7&org=57&genes=user&min_id=85&min_length=25&output=cns_mfa) at 89.3%

***** Conserved Regions - sequence1 Dmel_CrX_210000_330000 (sequence4 CV97L04) *****

42356 (99269) to 42400 (99313) = [45bp](http://pipeline.lbl.gov/cgi-bin/gp_cns?server=localhost&db=gp_server&run=u99-GXiIubQd&base=338&pos=Dmel_CrX_210000_330000:42356-42400&align_id=20&org=58&length=45&identity=91.1&type=intergenic&output=mfa) at 91.1% intergenic

43309 (105263) to 43334 (105288) = [26bp](http://pipeline.lbl.gov/cgi-bin/gp_cns?server=localhost&db=gp_server&run=u99-GXiIubQd&base=338&pos=Dmel_CrX_210000_330000:43309-43334&align_id=20&org=58&length=26&identity=88.5&type=exon&output=mfa) at 88.5% exon

43810 (105791) to 43834 (105815) = [25bp](http://pipeline.lbl.gov/cgi-bin/gp_cns?server=localhost&db=gp_server&run=u99-GXiIubQd&base=338&pos=Dmel_CrX_210000_330000:43810-43834&align_id=20&org=58&length=25&identity=88.0&type=exon&output=mfa) at 88.0% exon

Total [96bp](http://pipeline.lbl.gov/cgi-bin/gp_cns?server=localhost&db=gp_server&run=u99-GXiIubQd&base=338&pos=Dmel_CrX_210000_330000:1-120000&align_id=20&org=58&genes=user&min_id=85&min_length=25&output=cns_mfa) at 89.6%

***** Conserved Regions - sequence1 Dmel_CrX_210000_330000 (sequence5 CV62B24) *****

42356 (21655) to 42400 (21699) = [45bp](http://pipeline.lbl.gov/cgi-bin/gp_cns?server=localhost&db=gp_server&run=u99-GXiIubQd&base=338&pos=Dmel_CrX_210000_330000:42356-42400&align_id=3&org=59&length=45&identity=91.1&type=intergenic&output=mfa) at 91.1% intergenic

43309 (28144) to 43334 (28169) = [26bp](http://pipeline.lbl.gov/cgi-bin/gp_cns?server=localhost&db=gp_server&run=u99-GXiIubQd&base=338&pos=Dmel_CrX_210000_330000:43309-43334&align_id=3&org=59&length=26&identity=88.5&type=exon&output=mfa) at 88.5% exon

43810 (28672) to 43834 (28696) = [25bp](http://pipeline.lbl.gov/cgi-bin/gp_cns?server=localhost&db=gp_server&run=u99-GXiIubQd&base=338&pos=Dmel_CrX_210000_330000:43810-43834&align_id=3&org=59&length=25&identity=88.0&type=exon&output=mfa) at 88.0% exon

50554 (34567) to 50576 (34589) = [23bp](http://pipeline.lbl.gov/cgi-bin/gp_cns?server=localhost&db=gp_server&run=u99-GXiIubQd&base=338&pos=Dmel_CrX_210000_330000:50554-50576&align_id=3&org=59&length=23&identity=95.7&type=intergenic&output=mfa) at 95.7% intergenic

52919 (51925) to 52943 (51949) = [25bp](http://pipeline.lbl.gov/cgi-bin/gp_cns?server=localhost&db=gp_server&run=u99-GXiIubQd&base=338&pos=Dmel_CrX_210000_330000:52919-52943&align_id=3&org=59&length=25&identity=88.0&type=intergenic&output=mfa) at 88.0% intergenic

Total [144bp](http://pipeline.lbl.gov/cgi-bin/gp_cns?server=localhost&db=gp_server&run=u99-GXiIubQd&base=338&pos=Dmel_CrX_210000_330000:1-120000&align_id=3&org=59&genes=user&min_id=85&min_length=25&output=cns_mfa) at 90.3%

***** Conserved Regions - sequence1 Dmel_CrX_210000_330000 (sequence6 CV16B10) *****

56841 (49867) to 56869 (49895) = [29bp](http://pipeline.lbl.gov/cgi-bin/gp_cns?server=localhost&db=gp_server&run=u99-GXiIubQd&base=338&pos=Dmel_CrX_210000_330000:56841-56869&align_id=8&org=60&length=29&identity=89.7&type=exon&output=mfa) at 89.7% exon

57363 (50374) to 57388 (50399) = [26bp](http://pipeline.lbl.gov/cgi-bin/gp_cns?server=localhost&db=gp_server&run=u99-GXiIubQd&base=338&pos=Dmel_CrX_210000_330000:57363-57388&align_id=8&org=60&length=26&identity=92.3&type=exon&output=mfa) at 92.3% exon

Total [55bp](http://pipeline.lbl.gov/cgi-bin/gp_cns?server=localhost&db=gp_server&run=u99-GXiIubQd&base=338&pos=Dmel_CrX_210000_330000:1-120000&align_id=8&org=60&genes=user&min_id=85&min_length=25&output=cns_mfa) at 90.9%

***** Conserved Regions - sequence1 Dmel_CrX_210000_330000 (sequence7 104L14) *****

74581 (45845) to 74606 (45870) = [26bp](http://pipeline.lbl.gov/cgi-bin/gp_cns?server=localhost&db=gp_server&run=u99-GXiIubQd&base=338&pos=Dmel_CrX_210000_330000:74581-74606&align_id=4&org=61&length=26&identity=96.2&type=intergenic&output=mfa) at 96.2% intergenic

Total [26bp](http://pipeline.lbl.gov/cgi-bin/gp_cns?server=localhost&db=gp_server&run=u99-GXiIubQd&base=338&pos=Dmel_CrX_210000_330000:1-120000&align_id=4&org=61&genes=user&min_id=85&min_length=25&output=cns_mfa) at 96.2%

AVID

Calc Window, bp:

100

Min Cons Width, bp:

15

Cons Identity, %:

95

Minimum Y, %:

30

***** Conserved Regions - sequence1 Dmel_CrX_210000_330000 (sequence2 113H10) *****

13432 (18177) to 13450 (18195) = [19bp](http://pipeline.lbl.gov/cgi-bin/gp_cns?server=localhost&db=gp_server&run=u99-GXiIubQd&base=338&pos=Dmel_CrX_210000_330000:13432-13450&align_id=6&org=56&length=19&identity=100.0&type=intergenic&output=mfa) at 100.0% intergenic

Total [19bp](http://pipeline.lbl.gov/cgi-bin/gp_cns?server=localhost&db=gp_server&run=u99-GXiIubQd&base=338&pos=Dmel_CrX_210000_330000:1-120000&align_id=6&org=56&genes=user&min_id=95&min_length=15&output=cns_mfa) at 100.0%

***** Conserved Regions - sequence1 Dmel_CrX_210000_330000 (sequence3 CV99M22) *****

29770 (38767) to 29785 (38782) = [16bp](http://pipeline.lbl.gov/cgi-bin/gp_cns?server=localhost&db=gp_server&run=u99-GXiIubQd&base=338&pos=Dmel_CrX_210000_330000:29770-29785&align_id=7&org=57&length=16&identity=100.0&type=intergenic&output=mfa) at 100.0% intergenic

30576 (39066) to 30591 (39081) = [16bp](http://pipeline.lbl.gov/cgi-bin/gp_cns?server=localhost&db=gp_server&run=u99-GXiIubQd&base=338&pos=Dmel_CrX_210000_330000:30576-30591&align_id=7&org=57&length=16&identity=100.0&type=intergenic&output=mfa) at 100.0% intergenic

Total [32bp](http://pipeline.lbl.gov/cgi-bin/gp_cns?server=localhost&db=gp_server&run=u99-GXiIubQd&base=338&pos=Dmel_CrX_210000_330000:1-120000&align_id=7&org=57&genes=user&min_id=95&min_length=15&output=cns_mfa) at 100.0%

***** Conserved Regions - sequence1 Dmel_CrX_210000_330000 (sequence4 CV97L04) *****

42382 (99295) to 42400 (99313) = [19bp](http://pipeline.lbl.gov/cgi-bin/gp_cns?server=localhost&db=gp_server&run=u99-GXiIubQd&base=338&pos=Dmel_CrX_210000_330000:42382-42400&align_id=20&org=58&length=19&identity=100.0&type=intergenic&output=mfa) at 100.0% intergenic

42883 (104880) to 42897 (104894) = [15bp](http://pipeline.lbl.gov/cgi-bin/gp_cns?server=localhost&db=gp_server&run=u99-GXiIubQd&base=338&pos=Dmel_CrX_210000_330000:42883-42897&align_id=20&org=58&length=15&identity=100.0&type=UTR&output=mfa) at 100.0% UTR

Total [34bp](http://pipeline.lbl.gov/cgi-bin/gp_cns?server=localhost&db=gp_server&run=u99-GXiIubQd&base=338&pos=Dmel_CrX_210000_330000:1-120000&align_id=20&org=58&genes=user&min_id=95&min_length=15&output=cns_mfa) at 100.0%

***** Conserved Regions - sequence1 Dmel_CrX_210000_330000 (sequence5 CV62B24) *****

42382 (21681) to 42400 (21699) = [19bp](http://pipeline.lbl.gov/cgi-bin/gp_cns?server=localhost&db=gp_server&run=u99-GXiIubQd&base=338&pos=Dmel_CrX_210000_330000:42382-42400&align_id=3&org=59&length=19&identity=100.0&type=intergenic&output=mfa) at 100.0% intergenic

42883 (27765) to 42897 (27779) = [15bp](http://pipeline.lbl.gov/cgi-bin/gp_cns?server=localhost&db=gp_server&run=u99-GXiIubQd&base=338&pos=Dmel_CrX_210000_330000:42883-42897&align_id=3&org=59&length=15&identity=100.0&type=UTR&output=mfa) at 100.0% UTR

50560 (34573) to 50576 (34589) = [17bp](http://pipeline.lbl.gov/cgi-bin/gp_cns?server=localhost&db=gp_server&run=u99-GXiIubQd&base=338&pos=Dmel_CrX_210000_330000:50560-50576&align_id=3&org=59&length=17&identity=100.0&type=intergenic&output=mfa) at 100.0% intergenic

52927 (51933) to 52943 (51949) = [17bp](http://pipeline.lbl.gov/cgi-bin/gp_cns?server=localhost&db=gp_server&run=u99-GXiIubQd&base=338&pos=Dmel_CrX_210000_330000:52927-52943&align_id=3&org=59&length=17&identity=100.0&type=intergenic&output=mfa) at 100.0% intergenic

Total [68bp](http://pipeline.lbl.gov/cgi-bin/gp_cns?server=localhost&db=gp_server&run=u99-GXiIubQd&base=338&pos=Dmel_CrX_210000_330000:1-120000&align_id=3&org=59&genes=user&min_id=95&min_length=15&output=cns_mfa) at 100.0%

***** Conserved Regions - sequence1 Dmel_CrX_210000_330000 (sequence6 CV16B10) *****

22077 (34710) to 22092 (34725) = [16bp](http://pipeline.lbl.gov/cgi-bin/gp_cns?server=localhost&db=gp_server&run=u99-GXiIubQd&base=338&pos=Dmel_CrX_210000_330000:22077-22092&align_id=8&org=60&length=16&identity=100.0&type=intergenic&output=mfa) at 100.0% intergenic

57369 (50380) to 57385 (50396) = [17bp](http://pipeline.lbl.gov/cgi-bin/gp_cns?server=localhost&db=gp_server&run=u99-GXiIubQd&base=338&pos=Dmel_CrX_210000_330000:57369-57385&align_id=8&org=60&length=17&identity=100.0&type=exon&output=mfa) at 100.0% exon

57584 (50694) to 57600 (50710) = [17bp](http://pipeline.lbl.gov/cgi-bin/gp_cns?server=localhost&db=gp_server&run=u99-GXiIubQd&base=338&pos=Dmel_CrX_210000_330000:57584-57600&align_id=8&org=60&length=17&identity=100.0&type=UTR&output=mfa) at 100.0% UTR

Total [50bp](http://pipeline.lbl.gov/cgi-bin/gp_cns?server=localhost&db=gp_server&run=u99-GXiIubQd&base=338&pos=Dmel_CrX_210000_330000:1-120000&align_id=8&org=60&genes=user&min_id=95&min_length=15&output=cns_mfa) at 100.0%

***** Conserved Regions - sequence1 Dmel_CrX_210000_330000 (sequence7 104L14) *****

43000 (28651) to 43016 (28667) = [17bp](http://pipeline.lbl.gov/cgi-bin/gp_cns?server=localhost&db=gp_server&run=u99-GXiIubQd&base=338&pos=Dmel_CrX_210000_330000:43000-43016&align_id=4&org=61&length=17&identity=100.0&type=exon&output=mfa) at 100.0% exon

74581 (45845) to 74601 (45865) = [21bp](http://pipeline.lbl.gov/cgi-bin/gp_cns?server=localhost&db=gp_server&run=u99-GXiIubQd&base=338&pos=Dmel_CrX_210000_330000:74581-74601&align_id=4&org=61&length=21&identity=100.0&type=intergenic&output=mfa) at 100.0% intergenic

Total [38bp](http://pipeline.lbl.gov/cgi-bin/gp_cns?server=localhost&db=gp_server&run=u99-GXiIubQd&base=338&pos=Dmel_CrX_210000_330000:1-120000&align_id=4&org=61&genes=user&min_id=95&min_length=15&output=cns_mfa) at 100.0%

SLAGAN

Calc Window, bp:

100

Min Cons Width, bp:

100

Cons Identity, %:

70

Minimum Y, %:

50

Conserved intervals sorted by the sequence1 coordinates

***** Conserved Regions - sequence1 Dmel_CrX_210000_330000 (sequence2 113H10) *****

17004 (81057) to 17204 (81257) = [201bp](http://pipeline.lbl.gov/cgi-bin/gp_cns?server=localhost&db=gp_server&run=u100-Q6pJ5o7h&base=433&pos=Dmel_CrX_210000_330000:17004-17204&align_id=4&org=56&length=201&identity=69.7&type=exon&output=mfa) at 69.7% exon

[Get all CNS alignments](http://pipeline.lbl.gov/cgi-bin/gp_cns?server=localhost&db=gp_server&run=u100-Q6pJ5o7h&base=433&pos=Dmel_CrX_210000_330000:1-120000&org=56&genes=user&min_id=70&min_length=100&output=cns_mfa)

Conserved intervals sorted by the sequence1 coordinates

***** Conserved Regions - sequence1 Dmel_CrX_210000_330000 (sequence3 CV99M22) *****

17004 (22728) to 17204 (22928) = [201bp](http://pipeline.lbl.gov/cgi-bin/gp_cns?server=localhost&db=gp_server&run=u100-Q6pJ5o7h&base=433&pos=Dmel_CrX_210000_330000:17004-17204&align_id=166&org=57&length=201&identity=69.7&type=exon&output=mfa) at 69.7% exon

[Get all CNS alignments](http://pipeline.lbl.gov/cgi-bin/gp_cns?server=localhost&db=gp_server&run=u100-Q6pJ5o7h&base=433&pos=Dmel_CrX_210000_330000:1-120000&org=57&genes=user&min_id=70&min_length=100&output=cns_mfa)

***** Conserved Regions - sequence1 Dmel_CrX_210000_330000 (sequence4 CV97L04) *****

43285 (105239) to 43517 (105471) = [233bp](http://pipeline.lbl.gov/cgi-bin/gp_cns?server=localhost&db=gp_server&run=u100-Q6pJ5o7h&base=433&pos=Dmel_CrX_210000_330000:43285-43517&align_id=1&org=58&length=233&identity=73.8&type=exon&output=mfa) at 73.8% exon

43933 (105929) to 44035 (106031) = [103bp](http://pipeline.lbl.gov/cgi-bin/gp_cns?server=localhost&db=gp_server&run=u100-Q6pJ5o7h&base=433&pos=Dmel_CrX_210000_330000:43933-44035&align_id=1&org=58&length=103&identity=75.7&type=exon&output=mfa) at 75.7% exon

Total [336bp](http://pipeline.lbl.gov/cgi-bin/gp_cns?server=localhost&db=gp_server&run=u100-Q6pJ5o7h&base=433&pos=Dmel_CrX_210000_330000:17356-69037&align_id=1&org=58&genes=user&min_id=70&min_length=100&output=cns_mfa) at 74.4%

Conserved intervals sorted by the sequence1 coordinates

***** Conserved Regions - sequence1 Dmel_CrX_210000_330000 (sequence5 CV62B24) *****

43285 (28120) to 43517 (28352) = [233bp](http://pipeline.lbl.gov/cgi-bin/gp_cns?server=localhost&db=gp_server&run=u100-Q6pJ5o7h&base=433&pos=Dmel_CrX_210000_330000:43285-43517&align_id=48&org=59&length=233&identity=73.8&type=exon&output=mfa) at 73.8% exon

43933 (28810) to 44035 (28912) = [103bp](http://pipeline.lbl.gov/cgi-bin/gp_cns?server=localhost&db=gp_server&run=u100-Q6pJ5o7h&base=433&pos=Dmel_CrX_210000_330000:43933-44035&align_id=48&org=59&length=103&identity=76.7&type=exon&output=mfa) at 76.7% exon

[Get all CNS alignments](http://pipeline.lbl.gov/cgi-bin/gp_cns?server=localhost&db=gp_server&run=u100-Q6pJ5o7h&base=433&pos=Dmel_CrX_210000_330000:1-120000&org=59&genes=user&min_id=70&min_length=100&output=cns_mfa)

Conserved intervals sorted by the sequence1 coordinates

***** Conserved Regions - sequence1 Dmel_CrX_210000_330000 (sequence6 CV16B10) *****

52759 (24205) to 52864 (24089) = [117bp](http://pipeline.lbl.gov/cgi-bin/gp_cns?server=localhost&db=gp_server&run=u100-Q6pJ5o7h&base=433&pos=Dmel_CrX_210000_330000:52759-52864&align_id=28&org=60&length=117&identity=74.4&type=intergenic&output=mfa) at 74.4% intergenic

56786 (49818) to 57068 (50094) = [283bp](http://pipeline.lbl.gov/cgi-bin/gp_cns?server=localhost&db=gp_server&run=u100-Q6pJ5o7h&base=433&pos=Dmel_CrX_210000_330000:56786-57068&align_id=24&org=60&length=283&identity=73.1&type=exon&output=mfa) at 73.1% exon

[Get all CNS alignments](http://pipeline.lbl.gov/cgi-bin/gp_cns?server=localhost&db=gp_server&run=u100-Q6pJ5o7h&base=433&pos=Dmel_CrX_210000_330000:1-120000&org=60&genes=user&min_id=70&min_length=100&output=cns_mfa)

***** Conserved Regions - sequence1 Dmel_CrX_210000_330000 (sequence7 104L14) *****

Total [0bp](http://pipeline.lbl.gov/cgi-bin/gp_cns?server=localhost&db=gp_server&run=u100-Q6pJ5o7h&base=433&pos=Dmel_CrX_210000_330000:20692-120000&align_id=2&org=61&genes=user&min_id=70&min_length=100&output=cns_mfa) at 0.0%

SLAGAN

Calc Window, bp:

100

Min Cons Width, bp:

25

Cons Identity, %:

85

Minimum Y, %:

30

Conserved intervals sorted by the sequence1 coordinates

***** Conserved Regions - sequence1 Dmel_CrX_210000_330000 (sequence3 CV99M22) *****

56879 (22724) to 56914 (22759) = [36bp](http://pipeline.lbl.gov/cgi-bin/gp_cns?server=localhost&db=gp_server&run=u100-Q6pJ5o7h&base=433&pos=Dmel_CrX_210000_330000:56879-56914&align_id=162&org=57&length=36&identity=86.1&type=exon&output=mfa) at 86.1% exon

57579 (23749) to 57602 (23773) = [25bp](http://pipeline.lbl.gov/cgi-bin/gp_cns?server=localhost&db=gp_server&run=u100-Q6pJ5o7h&base=433&pos=Dmel_CrX_210000_330000:57579-57602&align_id=162&org=57&length=25&identity=92.0&type=UTR&output=mfa) at 92.0% UTR

[Get all CNS alignments](http://pipeline.lbl.gov/cgi-bin/gp_cns?server=localhost&db=gp_server&run=u100-Q6pJ5o7h&base=433&pos=Dmel_CrX_210000_330000:1-120000&org=57&genes=user&min_id=85&min_length=25&output=cns_mfa)

***** Conserved Regions - sequence1 Dmel_CrX_210000_330000 (sequence4 CV97L04) *****

42356 (99269) to 42400 (99313) = [45bp](http://pipeline.lbl.gov/cgi-bin/gp_cns?server=localhost&db=gp_server&run=u100-Q6pJ5o7h&base=433&pos=Dmel_CrX_210000_330000:42356-42400&align_id=1&org=58&length=45&identity=91.1&type=intergenic&output=mfa) at 91.1% intergenic

43309 (105263) to 43334 (105288) = [26bp](http://pipeline.lbl.gov/cgi-bin/gp_cns?server=localhost&db=gp_server&run=u100-Q6pJ5o7h&base=433&pos=Dmel_CrX_210000_330000:43309-43334&align_id=1&org=58&length=26&identity=88.5&type=exon&output=mfa) at 88.5% exon

43810 (105791) to 43834 (105815) = [25bp](http://pipeline.lbl.gov/cgi-bin/gp_cns?server=localhost&db=gp_server&run=u100-Q6pJ5o7h&base=433&pos=Dmel_CrX_210000_330000:43810-43834&align_id=1&org=58&length=25&identity=88.0&type=exon&output=mfa) at 88.0% exon

Total [96bp](http://pipeline.lbl.gov/cgi-bin/gp_cns?server=localhost&db=gp_server&run=u100-Q6pJ5o7h&base=433&pos=Dmel_CrX_210000_330000:17356-69037&align_id=1&org=58&genes=user&min_id=85&min_length=25&output=cns_mfa) at 89.6%

Conserved intervals sorted by the sequence1 coordinates

***** Conserved Regions - sequence1 Dmel_CrX_210000_330000 (sequence5 CV62B24) *****

42356 (21655) to 42400 (21699) = [45bp](http://pipeline.lbl.gov/cgi-bin/gp_cns?server=localhost&db=gp_server&run=u100-Q6pJ5o7h&base=433&pos=Dmel_CrX_210000_330000:42356-42400&align_id=48&org=59&length=45&identity=91.1&type=intergenic&output=mfa) at 91.1% intergenic

43309 (28144) to 43334 (28169) = [26bp](http://pipeline.lbl.gov/cgi-bin/gp_cns?server=localhost&db=gp_server&run=u100-Q6pJ5o7h&base=433&pos=Dmel_CrX_210000_330000:43309-43334&align_id=48&org=59&length=26&identity=88.5&type=exon&output=mfa) at 88.5% exon

43810 (28672) to 43834 (28696) = [25bp](http://pipeline.lbl.gov/cgi-bin/gp_cns?server=localhost&db=gp_server&run=u100-Q6pJ5o7h&base=433&pos=Dmel_CrX_210000_330000:43810-43834&align_id=48&org=59&length=25&identity=88.0&type=exon&output=mfa) at 88.0% exon

53863 (25146) to 53887 (25122) = [25bp](http://pipeline.lbl.gov/cgi-bin/gp_cns?server=localhost&db=gp_server&run=u100-Q6pJ5o7h&base=433&pos=Dmel_CrX_210000_330000:53863-53887&align_id=47&org=59&length=25&identity=88.0&type=intergenic&output=mfa) at 88.0% intergenic

[Get all CNS alignments](http://pipeline.lbl.gov/cgi-bin/gp_cns?server=localhost&db=gp_server&run=u100-Q6pJ5o7h&base=433&pos=Dmel_CrX_210000_330000:1-120000&org=59&genes=user&min_id=85&min_length=25&output=cns_mfa)

Conserved intervals sorted by the sequence1 coordinates

***** Conserved Regions - sequence1 Dmel_CrX_210000_330000 (sequence6 CV16B10) *****

51989 (30623) to 52013 (30599) = [25bp](http://pipeline.lbl.gov/cgi-bin/gp_cns?server=localhost&db=gp_server&run=u100-Q6pJ5o7h&base=433&pos=Dmel_CrX_210000_330000:51989-52013&align_id=28&org=60&length=25&identity=96.0&type=intergenic&output=mfa) at 96.0% intergenic

52800 (24163) to 52844 (24116) = [48bp](http://pipeline.lbl.gov/cgi-bin/gp_cns?server=localhost&db=gp_server&run=u100-Q6pJ5o7h&base=433&pos=Dmel_CrX_210000_330000:52800-52844&align_id=28&org=60&length=48&identity=87.5&type=intergenic&output=mfa) at 87.5% intergenic

53726 (19240) to 53759 (19207) = [34bp](http://pipeline.lbl.gov/cgi-bin/gp_cns?server=localhost&db=gp_server&run=u100-Q6pJ5o7h&base=433&pos=Dmel_CrX_210000_330000:53726-53759&align_id=28&org=60&length=34&identity=91.2&type=intergenic&output=mfa) at 91.2% intergenic

56841 (49867) to 56869 (49895) = [29bp](http://pipeline.lbl.gov/cgi-bin/gp_cns?server=localhost&db=gp_server&run=u100-Q6pJ5o7h&base=433&pos=Dmel_CrX_210000_330000:56841-56869&align_id=24&org=60&length=29&identity=89.7&type=exon&output=mfa) at 89.7% exon

57363 (50374) to 57388 (50399) = [26bp](http://pipeline.lbl.gov/cgi-bin/gp_cns?server=localhost&db=gp_server&run=u100-Q6pJ5o7h&base=433&pos=Dmel_CrX_210000_330000:57363-57388&align_id=24&org=60&length=26&identity=92.3&type=exon&output=mfa) at 92.3% exon

57584 (50694) to 57608 (50716) = [25bp](http://pipeline.lbl.gov/cgi-bin/gp_cns?server=localhost&db=gp_server&run=u100-Q6pJ5o7h&base=433&pos=Dmel_CrX_210000_330000:57584-57608&align_id=24&org=60&length=25&identity=88.0&type=UTR&output=mfa) at 88.0% UTR

[Get all CNS alignments](http://pipeline.lbl.gov/cgi-bin/gp_cns?server=localhost&db=gp_server&run=u100-Q6pJ5o7h&base=433&pos=Dmel_CrX_210000_330000:1-120000&org=60&genes=user&min_id=85&min_length=25&output=cns_mfa)

***** Conserved Regions - sequence1 Dmel_CrX_210000_330000 (sequence7 104L14) *****

69400 (32865) to 69427 (32892) = [28bp](http://pipeline.lbl.gov/cgi-bin/gp_cns?server=localhost&db=gp_server&run=u100-Q6pJ5o7h&base=433&pos=Dmel_CrX_210000_330000:69400-69427&align_id=2&org=61&length=28&identity=89.3&type=intergenic&output=mfa) at 89.3% intergenic

69638 (32987) to 69665 (33014) = [28bp](http://pipeline.lbl.gov/cgi-bin/gp_cns?server=localhost&db=gp_server&run=u100-Q6pJ5o7h&base=433&pos=Dmel_CrX_210000_330000:69638-69665&align_id=2&org=61&length=28&identity=96.4&type=intergenic&output=mfa) at 96.4% intergenic

74581 (45845) to 74601 (45865) = [21bp](http://pipeline.lbl.gov/cgi-bin/gp_cns?server=localhost&db=gp_server&run=u100-Q6pJ5o7h&base=433&pos=Dmel_CrX_210000_330000:74581-74601&align_id=2&org=61&length=21&identity=100.0&type=intergenic&output=mfa) at 100.0% intergenic

86922 (54520) to 86977 (54575) = [56bp](http://pipeline.lbl.gov/cgi-bin/gp_cns?server=localhost&db=gp_server&run=u100-Q6pJ5o7h&base=433&pos=Dmel_CrX_210000_330000:86922-86977&align_id=2&org=61&length=56&identity=87.5&type=intergenic&output=mfa) at 87.5% intergenic

90692 (69009) to 90719 (69036) = [28bp](http://pipeline.lbl.gov/cgi-bin/gp_cns?server=localhost&db=gp_server&run=u100-Q6pJ5o7h&base=433&pos=Dmel_CrX_210000_330000:90692-90719&align_id=2&org=61&length=28&identity=100.0&type=intergenic&output=mfa) at 100.0% intergenic

Total [161bp](http://pipeline.lbl.gov/cgi-bin/gp_cns?server=localhost&db=gp_server&run=u100-Q6pJ5o7h&base=433&pos=Dmel_CrX_210000_330000:20692-120000&align_id=2&org=61&genes=user&min_id=85&min_length=25&output=cns_mfa) at 93.2%

SLAGAN

Calc Window, bp:

100

Min Cons Width, bp:

15

Cons Identity, %:

95

Minimum Y, %:

30

Conserved intervals sorted by the sequence1 coordinates

***** Conserved Regions - sequence1 Dmel_CrX_210000_330000 (sequence2 113H10) *****

19402 (56321) to 19417 (56336) = [16bp](http://pipeline.lbl.gov/cgi-bin/gp_cns?server=localhost&db=gp_server&run=u100-Q6pJ5o7h&base=433&pos=Dmel_CrX_210000_330000:19402-19417&align_id=3&org=56&length=16&identity=100.0&type=intergenic&output=mfa) at 100.0% intergenic

[Get all CNS alignments](http://pipeline.lbl.gov/cgi-bin/gp_cns?server=localhost&db=gp_server&run=u100-Q6pJ5o7h&base=433&pos=Dmel_CrX_210000_330000:1-120000&org=56&genes=user&min_id=95&min_length=15&output=cns_mfa)

***** Conserved Regions - sequence1 Dmel_CrX_210000_330000 (sequence4 CV97L04) *****

42382 (99295) to 42400 (99313) = [19bp](http://pipeline.lbl.gov/cgi-bin/gp_cns?server=localhost&db=gp_server&run=u100-Q6pJ5o7h&base=433&pos=Dmel_CrX_210000_330000:42382-42400&align_id=1&org=58&length=19&identity=100.0&type=intergenic&output=mfa) at 100.0% intergenic

42883 (104880) to 42897 (104894) = [15bp](http://pipeline.lbl.gov/cgi-bin/gp_cns?server=localhost&db=gp_server&run=u100-Q6pJ5o7h&base=433&pos=Dmel_CrX_210000_330000:42883-42897&align_id=1&org=58&length=15&identity=100.0&type=UTR&output=mfa) at 100.0% UTR

Total [34bp](http://pipeline.lbl.gov/cgi-bin/gp_cns?server=localhost&db=gp_server&run=u100-Q6pJ5o7h&base=433&pos=Dmel_CrX_210000_330000:17356-69037&align_id=1&org=58&genes=user&min_id=95&min_length=15&output=cns_mfa) at 100.0%

Conserved intervals sorted by the sequence1 coordinates

***** Conserved Regions - sequence1 Dmel_CrX_210000_330000 (sequence5 CV62B24) *****

42382 (21681) to 42400 (21699) = [19bp](http://pipeline.lbl.gov/cgi-bin/gp_cns?server=localhost&db=gp_server&run=u100-Q6pJ5o7h&base=433&pos=Dmel_CrX_210000_330000:42382-42400&align_id=48&org=59&length=19&identity=100.0&type=intergenic&output=mfa) at 100.0% intergenic

42883 (27765) to 42897 (27779) = [15bp](http://pipeline.lbl.gov/cgi-bin/gp_cns?server=localhost&db=gp_server&run=u100-Q6pJ5o7h&base=433&pos=Dmel_CrX_210000_330000:42883-42897&align_id=48&org=59&length=15&identity=100.0&type=UTR&output=mfa) at 100.0% UTR

[Get all CNS alignments](http://pipeline.lbl.gov/cgi-bin/gp_cns?server=localhost&db=gp_server&run=u100-Q6pJ5o7h&base=433&pos=Dmel_CrX_210000_330000:1-120000&org=59&genes=user&min_id=95&min_length=15&output=cns_mfa)

Conserved intervals sorted by the sequence1 coordinates

***** Conserved Regions - sequence1 Dmel_CrX_210000_330000 (sequence6 CV16B10) *****

51989 (30623) to 52005 (30607) = [17bp](http://pipeline.lbl.gov/cgi-bin/gp_cns?server=localhost&db=gp_server&run=u100-Q6pJ5o7h&base=433&pos=Dmel_CrX_210000_330000:51989-52005&align_id=28&org=60&length=17&identity=100.0&type=intergenic&output=mfa) at 100.0% intergenic

52775 (24188) to 52792 (24171) = [18bp](http://pipeline.lbl.gov/cgi-bin/gp_cns?server=localhost&db=gp_server&run=u100-Q6pJ5o7h&base=433&pos=Dmel_CrX_210000_330000:52775-52792&align_id=28&org=60&length=18&identity=100.0&type=intergenic&output=mfa) at 100.0% intergenic

52804 (24159) to 52819 (24144) = [16bp](http://pipeline.lbl.gov/cgi-bin/gp_cns?server=localhost&db=gp_server&run=u100-Q6pJ5o7h&base=433&pos=Dmel_CrX_210000_330000:52804-52819&align_id=28&org=60&length=16&identity=100.0&type=intergenic&output=mfa) at 100.0% intergenic

53737 (19229) to 53759 (19207) = [23bp](http://pipeline.lbl.gov/cgi-bin/gp_cns?server=localhost&db=gp_server&run=u100-Q6pJ5o7h&base=433&pos=Dmel_CrX_210000_330000:53737-53759&align_id=28&org=60&length=23&identity=100.0&type=intergenic&output=mfa) at 100.0% intergenic

57369 (50380) to 57385 (50396) = [17bp](http://pipeline.lbl.gov/cgi-bin/gp_cns?server=localhost&db=gp_server&run=u100-Q6pJ5o7h&base=433&pos=Dmel_CrX_210000_330000:57369-57385&align_id=24&org=60&length=17&identity=100.0&type=exon&output=mfa) at 100.0% exon

57584 (50694) to 57600 (50710) = [17bp](http://pipeline.lbl.gov/cgi-bin/gp_cns?server=localhost&db=gp_server&run=u100-Q6pJ5o7h&base=433&pos=Dmel_CrX_210000_330000:57584-57600&align_id=24&org=60&length=17&identity=100.0&type=UTR&output=mfa) at 100.0% UTR

[Get all CNS alignments](http://pipeline.lbl.gov/cgi-bin/gp_cns?server=localhost&db=gp_server&run=u100-Q6pJ5o7h&base=433&pos=Dmel_CrX_210000_330000:1-120000&org=60&genes=user&min_id=95&min_length=15&output=cns_mfa)

***** Conserved Regions - sequence1 Dmel_CrX_210000_330000 (sequence7 104L14) *****

50555 (28649) to 50573 (28667) = [19bp](http://pipeline.lbl.gov/cgi-bin/gp_cns?server=localhost&db=gp_server&run=u100-Q6pJ5o7h&base=433&pos=Dmel_CrX_210000_330000:50555-50573&align_id=2&org=61&length=19&identity=100.0&type=intergenic&output=mfa) at 100.0% intergenic

69405 (32870) to 69423 (32888) = [19bp](http://pipeline.lbl.gov/cgi-bin/gp_cns?server=localhost&db=gp_server&run=u100-Q6pJ5o7h&base=433&pos=Dmel_CrX_210000_330000:69405-69423&align_id=2&org=61&length=19&identity=100.0&type=intergenic&output=mfa) at 100.0% intergenic

69643 (32992) to 69665 (33014) = [23bp](http://pipeline.lbl.gov/cgi-bin/gp_cns?server=localhost&db=gp_server&run=u100-Q6pJ5o7h&base=433&pos=Dmel_CrX_210000_330000:69643-69665&align_id=2&org=61&length=23&identity=100.0&type=intergenic&output=mfa) at 100.0% intergenic

74581 (45845) to 74601 (45865) = [21bp](http://pipeline.lbl.gov/cgi-bin/gp_cns?server=localhost&db=gp_server&run=u100-Q6pJ5o7h&base=433&pos=Dmel_CrX_210000_330000:74581-74601&align_id=2&org=61&length=21&identity=100.0&type=intergenic&output=mfa) at 100.0% intergenic

86953 (54551) to 86968 (54566) = [16bp](http://pipeline.lbl.gov/cgi-bin/gp_cns?server=localhost&db=gp_server&run=u100-Q6pJ5o7h&base=433&pos=Dmel_CrX_210000_330000:86953-86968&align_id=2&org=61&length=16&identity=100.0&type=intergenic&output=mfa) at 100.0% intergenic

90692 (69009) to 90719 (69036) = [28bp](http://pipeline.lbl.gov/cgi-bin/gp_cns?server=localhost&db=gp_server&run=u100-Q6pJ5o7h&base=433&pos=Dmel_CrX_210000_330000:90692-90719&align_id=2&org=61&length=28&identity=100.0&type=intergenic&output=mfa) at 100.0% intergenic

Total [126bp](http://pipeline.lbl.gov/cgi-bin/gp_cns?server=localhost&db=gp_server&run=u100-Q6pJ5o7h&base=433&pos=Dmel_CrX_210000_330000:20692-120000&align_id=2&org=61&genes=user&min_id=95&min_length=15&output=cns_mfa) at 100.0%

MLAGAN

Calc Window, bp:

100

Min Cons Width, bp:

100

Cons Identity, %:

70

Minimum Y, %:

50

***** Conserved Regions - sequence1 Dmel_CrX_210000_330000 (sequence2 113H10) *****

17004 (81057) to 17204 (81257) = [201bp](http://pipeline.lbl.gov/cgi-bin/gp_cns?server=localhost&db=gp_server&run=u98-BVzmp9AF&base=331&pos=Dmel_CrX_210000_330000:17004-17204&align_id=1&org=56&length=201&identity=69.7&type=exon&output=mfa) at 69.7% exon

Total [201bp](http://pipeline.lbl.gov/cgi-bin/gp_cns?server=localhost&db=gp_server&run=u98-BVzmp9AF&base=331&pos=Dmel_CrX_210000_330000:1-120000&align_id=1&org=56&genes=user&min_id=70&min_length=100&output=cns_mfa) at 69.7%

***** Conserved Regions - sequence1 Dmel_CrX_210000_330000 (sequence3 CV99M22) *****

17004 (22728) to 17204 (22928) = [201bp](http://pipeline.lbl.gov/cgi-bin/gp_cns?server=localhost&db=gp_server&run=u98-BVzmp9AF&base=331&pos=Dmel_CrX_210000_330000:17004-17204&align_id=1&org=57&length=201&identity=69.7&type=exon&output=mfa) at 69.7% exon

Total [201bp](http://pipeline.lbl.gov/cgi-bin/gp_cns?server=localhost&db=gp_server&run=u98-BVzmp9AF&base=331&pos=Dmel_CrX_210000_330000:1-120000&align_id=1&org=57&genes=user&min_id=70&min_length=100&output=cns_mfa) at 69.7%

***** Conserved Regions - sequence1 Dmel_CrX_210000_330000 (sequence4 CV97L04) *****

43285 (105239) to 43511 (105465) = [227bp](http://pipeline.lbl.gov/cgi-bin/gp_cns?server=localhost&db=gp_server&run=u98-BVzmp9AF&base=331&pos=Dmel_CrX_210000_330000:43285-43511&align_id=1&org=58&length=227&identity=73.6&type=exon&output=mfa) at 73.6% exon

43933 (105929) to 44035 (106031) = [103bp](http://pipeline.lbl.gov/cgi-bin/gp_cns?server=localhost&db=gp_server&run=u98-BVzmp9AF&base=331&pos=Dmel_CrX_210000_330000:43933-44035&align_id=1&org=58&length=103&identity=75.7&type=exon&output=mfa) at 75.7% exon

Total [330bp](http://pipeline.lbl.gov/cgi-bin/gp_cns?server=localhost&db=gp_server&run=u98-BVzmp9AF&base=331&pos=Dmel_CrX_210000_330000:1-120000&align_id=1&org=58&genes=user&min_id=70&min_length=100&output=cns_mfa) at 74.2%

***** Conserved Regions - sequence1 Dmel_CrX_210000_330000 (sequence5 CV62B24) *****

43285 (28120) to 43511 (28346) = [227bp](http://pipeline.lbl.gov/cgi-bin/gp_cns?server=localhost&db=gp_server&run=u98-BVzmp9AF&base=331&pos=Dmel_CrX_210000_330000:43285-43511&align_id=1&org=59&length=227&identity=73.6&type=exon&output=mfa) at 73.6% exon

43933 (28810) to 44035 (28912) = [103bp](http://pipeline.lbl.gov/cgi-bin/gp_cns?server=localhost&db=gp_server&run=u98-BVzmp9AF&base=331&pos=Dmel_CrX_210000_330000:43933-44035&align_id=1&org=59&length=103&identity=76.7&type=exon&output=mfa) at 76.7% exon

Total [330bp](http://pipeline.lbl.gov/cgi-bin/gp_cns?server=localhost&db=gp_server&run=u98-BVzmp9AF&base=331&pos=Dmel_CrX_210000_330000:1-120000&align_id=1&org=59&genes=user&min_id=70&min_length=100&output=cns_mfa) at 74.5%

***** Conserved Regions - sequence1 Dmel_CrX_210000_330000 (sequence6 CV16B10) *****

56820 (49846) to 57055 (50081) = [236bp](http://pipeline.lbl.gov/cgi-bin/gp_cns?server=localhost&db=gp_server&run=u98-BVzmp9AF&base=331&pos=Dmel_CrX_210000_330000:56820-57055&align_id=1&org=60&length=236&identity=76.3&type=exon&output=mfa) at 76.3% exon

Total [236bp](http://pipeline.lbl.gov/cgi-bin/gp_cns?server=localhost&db=gp_server&run=u98-BVzmp9AF&base=331&pos=Dmel_CrX_210000_330000:1-120000&align_id=1&org=60&genes=user&min_id=70&min_length=100&output=cns_mfa) at 76.3%

***** Conserved Regions - sequence1 Dmel_CrX_210000_330000 (sequence7 104L14) *****

Total [0bp](http://pipeline.lbl.gov/cgi-bin/gp_cns?server=localhost&db=gp_server&run=u98-BVzmp9AF&base=331&pos=Dmel_CrX_210000_330000:1-120000&align_id=1&org=61&genes=user&min_id=70&min_length=100&output=cns_mfa) at 0.0%

MLAGAN

Calc Window, bp:

100

Min Cons Width, bp:

25

Cons Identity, %:

85

Minimum Y, %:

30

***** Conserved Regions - sequence1 Dmel_CrX_210000_330000 (sequence2 113H10) *****

Total [0bp](http://pipeline.lbl.gov/cgi-bin/gp_cns?server=localhost&db=gp_server&run=u98-BVzmp9AF&base=331&pos=Dmel_CrX_210000_330000:1-120000&align_id=1&org=56&genes=user&min_id=85&min_length=25&output=cns_mfa) at 0.0%

***** Conserved Regions - sequence1 Dmel_CrX_210000_330000 (sequence3 CV99M22) *****

Total [0bp](http://pipeline.lbl.gov/cgi-bin/gp_cns?server=localhost&db=gp_server&run=u98-BVzmp9AF&base=331&pos=Dmel_CrX_210000_330000:1-120000&align_id=1&org=57&genes=user&min_id=85&min_length=25&output=cns_mfa) at 0.0%

***** Conserved Regions - sequence1 Dmel_CrX_210000_330000 (sequence4 CV97L04) *****

42356 (99269) to 42400 (99313) = [45bp](http://pipeline.lbl.gov/cgi-bin/gp_cns?server=localhost&db=gp_server&run=u98-BVzmp9AF&base=331&pos=Dmel_CrX_210000_330000:42356-42400&align_id=1&org=58&length=45&identity=91.1&type=intergenic&output=mfa) at 91.1% intergenic

43309 (105263) to 43334 (105288) = [26bp](http://pipeline.lbl.gov/cgi-bin/gp_cns?server=localhost&db=gp_server&run=u98-BVzmp9AF&base=331&pos=Dmel_CrX_210000_330000:43309-43334&align_id=1&org=58&length=26&identity=88.5&type=exon&output=mfa) at 88.5% exon

43810 (105791) to 43834 (105815) = [25bp](http://pipeline.lbl.gov/cgi-bin/gp_cns?server=localhost&db=gp_server&run=u98-BVzmp9AF&base=331&pos=Dmel_CrX_210000_330000:43810-43834&align_id=1&org=58&length=25&identity=88.0&type=exon&output=mfa) at 88.0% exon

Total [96bp](http://pipeline.lbl.gov/cgi-bin/gp_cns?server=localhost&db=gp_server&run=u98-BVzmp9AF&base=331&pos=Dmel_CrX_210000_330000:1-120000&align_id=1&org=58&genes=user&min_id=85&min_length=25&output=cns_mfa) at 89.6%

***** Conserved Regions - sequence1 Dmel_CrX_210000_330000 (sequence5 CV62B24) *****

42356 (21655) to 42400 (21699) = [45bp](http://pipeline.lbl.gov/cgi-bin/gp_cns?server=localhost&db=gp_server&run=u98-BVzmp9AF&base=331&pos=Dmel_CrX_210000_330000:42356-42400&align_id=1&org=59&length=45&identity=91.1&type=intergenic&output=mfa) at 91.1% intergenic

43309 (28144) to 43334 (28169) = [26bp](http://pipeline.lbl.gov/cgi-bin/gp_cns?server=localhost&db=gp_server&run=u98-BVzmp9AF&base=331&pos=Dmel_CrX_210000_330000:43309-43334&align_id=1&org=59&length=26&identity=88.5&type=exon&output=mfa) at 88.5% exon

43810 (28672) to 43834 (28696) = [25bp](http://pipeline.lbl.gov/cgi-bin/gp_cns?server=localhost&db=gp_server&run=u98-BVzmp9AF&base=331&pos=Dmel_CrX_210000_330000:43810-43834&align_id=1&org=59&length=25&identity=88.0&type=exon&output=mfa) at 88.0% exon

Total [96bp](http://pipeline.lbl.gov/cgi-bin/gp_cns?server=localhost&db=gp_server&run=u98-BVzmp9AF&base=331&pos=Dmel_CrX_210000_330000:1-120000&align_id=1&org=59&genes=user&min_id=85&min_length=25&output=cns_mfa) at 89.6%

***** Conserved Regions - sequence1 Dmel_CrX_210000_330000 (sequence6 CV16B10) *****

56841 (49867) to 56869 (49895) = [29bp](http://pipeline.lbl.gov/cgi-bin/gp_cns?server=localhost&db=gp_server&run=u98-BVzmp9AF&base=331&pos=Dmel_CrX_210000_330000:56841-56869&align_id=1&org=60&length=29&identity=89.7&type=exon&output=mfa) at 89.7% exon

57363 (50374) to 57388 (50399) = [26bp](http://pipeline.lbl.gov/cgi-bin/gp_cns?server=localhost&db=gp_server&run=u98-BVzmp9AF&base=331&pos=Dmel_CrX_210000_330000:57363-57388&align_id=1&org=60&length=26&identity=92.3&type=exon&output=mfa) at 92.3% exon

Total [55bp](http://pipeline.lbl.gov/cgi-bin/gp_cns?server=localhost&db=gp_server&run=u98-BVzmp9AF&base=331&pos=Dmel_CrX_210000_330000:1-100000&align_id=1&org=60&genes=user&min_id=85&min_length=25&output=cns_mfa) at 90.9%

***** Conserved Regions - sequence1 Dmel_CrX_210000_330000 (sequence7 104L14) *****

69397 (32862) to 69423 (32888) = [27bp](http://pipeline.lbl.gov/cgi-bin/gp_cns?server=localhost&db=gp_server&run=u98-BVzmp9AF&base=331&pos=Dmel_CrX_210000_330000:69397-69423&align_id=1&org=61&length=27&identity=88.9&type=intergenic&output=mfa) at 88.9% intergenic

69638 (32987) to 69665 (33014) = [28bp](http://pipeline.lbl.gov/cgi-bin/gp_cns?server=localhost&db=gp_server&run=u98-BVzmp9AF&base=331&pos=Dmel_CrX_210000_330000:69638-69665&align_id=1&org=61&length=28&identity=96.4&type=intergenic&output=mfa) at 96.4% intergenic

86922 (54520) to 86976 (54574) = [55bp](http://pipeline.lbl.gov/cgi-bin/gp_cns?server=localhost&db=gp_server&run=u98-BVzmp9AF&base=331&pos=Dmel_CrX_210000_330000:86922-86976&align_id=1&org=61&length=55&identity=87.3&type=intergenic&output=mfa) at 87.3% intergenic

90692 (69009) to 90719 (69036) = [28bp](http://pipeline.lbl.gov/cgi-bin/gp_cns?server=localhost&db=gp_server&run=u98-BVzmp9AF&base=331&pos=Dmel_CrX_210000_330000:90692-90719&align_id=1&org=61&length=28&identity=100.0&type=intergenic&output=mfa) at 100.0% intergenic

Total [138bp](http://pipeline.lbl.gov/cgi-bin/gp_cns?server=localhost&db=gp_server&run=u98-BVzmp9AF&base=331&pos=Dmel_CrX_210000_330000:1-120000&align_id=1&org=61&genes=user&min_id=85&min_length=25&output=cns_mfa) at 92.0%

MLAGAN

Calc Window, bp:

100

Min Cons Width, bp:

15

Cons Identity, %:

95

Minimum Y, %:

30

***** Conserved Regions - sequence1 Dmel_CrX_210000_330000 (sequence2 113H10) *****

Total [0bp](http://pipeline.lbl.gov/cgi-bin/gp_cns?server=localhost&db=gp_server&run=u98-BVzmp9AF&base=331&pos=Dmel_CrX_210000_330000:1-120000&align_id=1&org=56&genes=user&min_id=95&min_length=15&output=cns_mfa) at 0.0%

***** Conserved Regions - sequence1 Dmel_CrX_210000_330000 (sequence3 CV99M22) *****

Total [0bp](http://pipeline.lbl.gov/cgi-bin/gp_cns?server=localhost&db=gp_server&run=u98-BVzmp9AF&base=331&pos=Dmel_CrX_210000_330000:1-120000&align_id=1&org=57&genes=user&min_id=95&min_length=15&output=cns_mfa) at 0.0%

***** Conserved Regions - sequence1 Dmel_CrX_210000_330000 (sequence4 CV97L04) *****

42382 (99295) to 42400 (99313) = [19bp](http://pipeline.lbl.gov/cgi-bin/gp_cns?server=localhost&db=gp_server&run=u98-BVzmp9AF&base=331&pos=Dmel_CrX_210000_330000:42382-42400&align_id=1&org=58&length=19&identity=100.0&type=intergenic&output=mfa) at 100.0% intergenic

42883 (104880) to 42897 (104894) = [15bp](http://pipeline.lbl.gov/cgi-bin/gp_cns?server=localhost&db=gp_server&run=u98-BVzmp9AF&base=331&pos=Dmel_CrX_210000_330000:42883-42897&align_id=1&org=58&length=15&identity=100.0&type=UTR&output=mfa) at 100.0% UTR

Total [34bp](http://pipeline.lbl.gov/cgi-bin/gp_cns?server=localhost&db=gp_server&run=u98-BVzmp9AF&base=331&pos=Dmel_CrX_210000_330000:1-120000&align_id=1&org=58&genes=user&min_id=95&min_length=15&output=cns_mfa) at 100.0%

***** Conserved Regions - sequence1 Dmel_CrX_210000_330000 (sequence5 CV62B24) *****

42382 (21681) to 42400 (21699) = [19bp](http://pipeline.lbl.gov/cgi-bin/gp_cns?server=localhost&db=gp_server&run=u98-BVzmp9AF&base=331&pos=Dmel_CrX_210000_330000:42382-42400&align_id=1&org=59&length=19&identity=100.0&type=intergenic&output=mfa) at 100.0% intergenic

42883 (27765) to 42897 (27779) = [15bp](http://pipeline.lbl.gov/cgi-bin/gp_cns?server=localhost&db=gp_server&run=u98-BVzmp9AF&base=331&pos=Dmel_CrX_210000_330000:42883-42897&align_id=1&org=59&length=15&identity=100.0&type=UTR&output=mfa) at 100.0% UTR

Total [34bp](http://pipeline.lbl.gov/cgi-bin/gp_cns?server=localhost&db=gp_server&run=u98-BVzmp9AF&base=331&pos=Dmel_CrX_210000_330000:1-120000&align_id=1&org=59&genes=user&min_id=95&min_length=15&output=cns_mfa) at 100.0%

***** Conserved Regions - sequence1 Dmel_CrX_210000_330000 (sequence6 CV16B10) *****

57369 (50380) to 57385 (50396) = [17bp](http://pipeline.lbl.gov/cgi-bin/gp_cns?server=localhost&db=gp_server&run=u98-BVzmp9AF&base=331&pos=Dmel_CrX_210000_330000:57369-57385&align_id=1&org=60&length=17&identity=100.0&type=exon&output=mfa) at 100.0% exon

57584 (50694) to 57600 (50710) = [17bp](http://pipeline.lbl.gov/cgi-bin/gp_cns?server=localhost&db=gp_server&run=u98-BVzmp9AF&base=331&pos=Dmel_CrX_210000_330000:57584-57600&align_id=1&org=60&length=17&identity=100.0&type=UTR&output=mfa) at 100.0% UTR

Total [34bp](http://pipeline.lbl.gov/cgi-bin/gp_cns?server=localhost&db=gp_server&run=u98-BVzmp9AF&base=331&pos=Dmel_CrX_210000_330000:1-120000&align_id=1&org=60&genes=user&min_id=95&min_length=15&output=cns_mfa) at 100.0%

***** Conserved Regions - sequence1 Dmel_CrX_210000_330000 (sequence7 104L14) *****

69405 (32870) to 69423 (32888) = [19bp](http://pipeline.lbl.gov/cgi-bin/gp_cns?server=localhost&db=gp_server&run=u98-BVzmp9AF&base=331&pos=Dmel_CrX_210000_330000:69405-69423&align_id=1&org=61&length=19&identity=100.0&type=intergenic&output=mfa) at 100.0% intergenic

69643 (32992) to 69665 (33014) = [23bp](http://pipeline.lbl.gov/cgi-bin/gp_cns?server=localhost&db=gp_server&run=u98-BVzmp9AF&base=331&pos=Dmel_CrX_210000_330000:69643-69665&align_id=1&org=61&length=23&identity=100.0&type=intergenic&output=mfa) at 100.0% intergenic

86953 (54551) to 86968 (54566) = [16bp](http://pipeline.lbl.gov/cgi-bin/gp_cns?server=localhost&db=gp_server&run=u98-BVzmp9AF&base=331&pos=Dmel_CrX_210000_330000:86953-86968&align_id=1&org=61&length=16&identity=100.0&type=intergenic&output=mfa) at 100.0% intergenic

90692 (69009) to 90719 (69036) = [28bp](http://pipeline.lbl.gov/cgi-bin/gp_cns?server=localhost&db=gp_server&run=u98-BVzmp9AF&base=331&pos=Dmel_CrX_210000_330000:90692-90719&align_id=1&org=61&length=28&identity=100.0&type=intergenic&output=mfa) at 100.0% intergenic

Total [86bp](http://pipeline.lbl.gov/cgi-bin/gp_cns?server=localhost&db=gp_server&run=u98-BVzmp9AF&base=331&pos=Dmel_CrX_210000_330000:1-120000&align_id=1&org=61&genes=user&min_id=95&min_length=15&output=cns_mfa) at 100.0%
